# Supplementary material for: Incommensurate Structural and Magnetic Modulations in Potassium-Rich Cryptomelane, K x Mn8O16 (x ≈ 1.45)
Source: Chem Mater. 2026 Oct 6;38(20):10711–25. doi: 10.1021/acs.chemmater.6c01980 (PMC13718541; doi:10.1021/acs.chemmater.6c01980)
Supplement: Supplementary file 1 [file cm6c01980_si_001.pdf]

Incommensurate structural and magnetic modulations in potassium-rich cryptomelane, $K_xMn_8O_{16}$ ($x \approx 1.45$): SUPPLEMENTARY MATERIAL

Liam A. V. Nagle-Cocco [a], [1,2,3] Joshua D. Bocarsly [b], [1,2] Krishnakanth Sada [c], [4,1] Nicola D. Kelly,[1] Mathias A. Kiefer,[3] Emmanuelle Suard,[5] Sarah J. Day,[6] Cheng Liu,[1] Clare P. Grey,[2] Prabeer Barpanda,[4] Clemens Ritter,[5] and Siân E. Dutton [d] [1]

[1] *Cavendish Laboratory, University of Cambridge, Cambridge, CB3 0US, United Kingdom.*

[2] *Yusuf Hamied Department of Chemistry, University of Cambridge, Cambridge, CB2 1EW, United Kingdom.*

[3] *Stanford Synchrotron Radiation Lightsource, SLAC National Accelerator Laboratory, Menlo Park, California 94025, United States.*

[4] *Faraday Materials Laboratory (FaMaL), Materials Research Centre, Indian Institute of Science, C. V. Raman Avenue, Bangalore, 560012, India.*

[5] *Institut Laue-Langevin, 71 avenue des Martyrs, CS 20156, cedex 9, Grenoble 38042, France.*

[6] *Diamond Light Source, Ltd., Harwell Science and Innovation Campus, Didcot, Oxfordshire OX11 0DE, United Kingdom.*

[a] Email: lnc@stanford.edu

[b] Present address: Department of Chemistry *and* Texas Center for Superconductivity, University of Houston, Houston, Texas 77004, United States of America.

[c] Present address: Xerion Advanced Battery Corporation, Kettering, Ohio 45420, United States of America.

[d] Email: sed33@cam.ac.uk

CONTENTS

List of Figures S-4

List of Tables S-6

1. Measurement sample summary S-7

2. Scanning Electron Microscopy S-9

3. Calculating magnetic heat capacity S-10

4. Low-temperature powder diffraction S-12
   4.1. Neutron powder diffraction S-12
   4.2. Laboratory X-ray diffraction S-17
   4.3. Low-temperature synchrotron X-ray diffraction S-20
      4.3.1. Introduction S-20
      4.3.2. Sequential refinement of synchrotron data: monoclinic cell S-24
      4.3.3. Sequential refinement of synchrotron data: tetragonal cell S-39
      4.3.4. Indexing of the incommensurate structural propagation vector S-41

5. Octahedral distortion parameters S-44
   5.1. Bond length distortion index S-44
   5.2. Bond angle variance S-44
   5.3. Q_3 mode S-44

6. AC Susceptibility: derivative with temperature S-45

7. Magnetic models used for neutron diffraction analysis S-47
   7.1. Monoclinic IRs S-47
   7.2. Tetragonal IRs S-50
   7.3. Phase shifts S-50

8. Magnetic neutron diffraction analysis for $T_3 < T < T_2$ assuming monoclinic cell S-53
   8.1. Varying incommensurate models at 47 K S-53
   8.2. Comparison to fits at 40 K and 44 K S-57

9. Magnetic neutron diffraction analysis at 47 K assuming tetragonal cell S-60

10. Magnetometry on KMO-S2 S-66

11. Curie-Weiss on KMO-S1 S-68

11.1. Error propagation S-68

References S-70

LIST OF FIGURES

S1 Electron microscope images of cryptomelane particles S-9
S2 Obtaining C_{mag} from experimental heat capacity S-11
S3 Low-temperature powder neutron diffraction measurements S-13
S4 Low-temperature powder neutron diffraction difference patterns S-14
S5 Neutron diffraction peak intensities with temperature S-15
S6 Low-temperature non-isothermal powder neutron diffraction S-16
S7 Low-temperature powder X-ray diffraction measurement on cryptomelane . . . S-17
S8 Rietveld refinement results for low-temperature ($T < 60$ K) laboratory powder X-ray diffraction S-18
S9 Comparison of synchrotron data at various temperatures for KMO-S2 . . . . . S-21
S10 Heat map of synchrotron diffraction data for KMO-S2 . . . . . S-22
S11 Example fits in the sequential Rietveld refinement on KMO-S2 . . . . . S-25
S12 Refined unit cell parameters against temperature for monoclinic cryptomelane between 100 K and 300 K S-26
S13 Refined pseudo-tetragonal unit cell parameters against temperature for monoclinic cryptomelane between 100 K and 300 K S-27
S14 Refined relative pseudo-tetragonal unit cell parameters against temperature for monoclinic cryptomelane between 100 K and 300 K S-28
S15 Refined unit cell volume against temperature for cryptomelane between 100 K and 300 K S-29
S16 Refined Stephens' parameters against temperature for monoclinic cryptomelane between 100 K and 300 K S-30
S17 Refined ADPs against temperature for monoclinic cryptomelane between 100 K and 300 K S-31
S18 Mn positions against temperature from sequential Rietveld refinement for monoclinic cryptomelane between 100 K and 300 K S-32
S19 K position against temperature from sequential Rietveld refinement for monoclinic cryptomelane between 100 K and 300 K S-33
S20 Mn–O bond lengths against temperature from sequential Rietveld refinement of synchrotron diffraction data S-34

S21 MnO_6 octahedral distortion against temperature from sequential Rietveld refinement of synchrotron diffraction data . . . S-35
S22 Equatorial octahedral O–O distances against temperature from sequential Rietveld refinement of synchrotron diffraction data . . . S-36
S23 Bond valence sum against temperature for the two Mn sites from sequential Rietveld refinement of synchrotron diffraction data . . . S-37
S24 R_{wp} against temperature for the sequential Rietveld refinement fits . . . S-38
S25 Refined unit cell parameters against temperature for tetragonal cryptomelane between 100 K and 300 K . . . S-40
S26 Joint fit of the four satellite reflections used to determine k_z . . . S-42
S27 Temperature dependence of the structural propagation vector . . . S-43
S28 AC susceptibility: derivative with temperature . . . S-45
S29 Rietveld fit of sinusoidal model to magnetic neutron diffraction data at 47 K assuming monoclinic cell . . . S-54
S30 Magnetic structure at 47 K assuming monoclinic cell . . . S-55
S31 Rietveld fit of ellipsoidal helix model to magnetic neutron diffraction data at 47 K assuming monoclinic cell . . . S-56
S32 Rietveld fit of helical model to magnetic neutron diffraction data at 44 K assuming monoclinic cell . . . S-58
S33 Rietveld fit of helical model to magnetic neutron diffraction data at 40 K assuming monoclinic cell . . . S-59
S34 Rietveld fit of helix model to magnetic neutron diffraction data at 47 K assuming tetragonal cell . . . S-62
S35 Magnetic structures at 47 K assuming tetragonal cell . . . S-64
S36 Possible commensurate components of the tetragonal magnetic structure at 47 K . . . S-65
S37 Magnetometry on second cryptomelane sample . . . S-67

LIST OF TABLES

S1 Summary of characterization techniques for both cryptomelane batches studied in this work S-7

S2 Room-temperature structural and phase comparison of two cryptomelane batches S-8

S3 Lattice parameters of cryptomelane at low temperatures from lab XRD S-19

S4 Crystallographic parameters of monoclinic cryptomelane at 100 K S-23

S5 Irreducible representations (monoclinic cell) for $\vec{k}_{\text{mag}} = (0, 0.37, 0)$ S-48

S6 Irreducible representations (monoclinic cell) for $\vec{k}_{\text{mag}} = (0, 0, 0)$ S-49

S7 Irreducible representations (tetragonal cell) for $\vec{k}_{\text{mag}} = (0, 0, 0.37)$ S-51

S8 Irreducible representations (tetragonal cell) for $\vec{k}_{\text{mag}} = (0, 0, 0)$ S-52

S9 Refined moment magnitudes at 47, 44, and 40 K S-57

S10 Structural and magnetometric transition temperatures for KMO-S1 and KMO-S2 . . . . . S-66

1. MEASUREMENT SAMPLE SUMMARY

In this paper, we studied two separately-synthesized batches of cryptomelane, denoted KMO-S1 and KMO-S2, both prepared using the identical solid-state route and target composition described in Methods. Both were synthesized in the same way, but due to the high sensitivity of material properties to sample stoichiometry, we tabulate in Table S1 which measurements were performed on each sample. Table S2 shows the room-temperature lattice parameters and impurity phase fractions of the two samples. Combined neutron and synchrotron Rietveld refinement, performed only for KMO-S1, allowed its composition to be precisely refined; for KMO-S2, which was characterized only by synchrotron diffraction, refined K occupancy is not independent of correlated atomic displacement, strain, and impurity scale-factor parameters, so we cannot refine K content to the same level of precision. Table S2 shows that they have different secondary-phase weight fractions, and they also have different T_1 transition temperatures (Section 10, and Section 4.3 of this Supplementary Information; see also the main text). However, the two batches have closely comparable room-temperature lattice parameters for the cryptomelane phase (Table S2), to which the tunnel K content has been shown to be highly sensitive, indicating a similar K content in both batches despite some batch-to-batch variation.

TABLE S1: Summary of characterization techniques for both cryptomelane batches studied in this work. RT is room temperature.

	**KMO-S1**	**KMO-S2**
Synchrotron XRD	RT only	Variable-temperature (100 K to 300 K)
Low-T lab XRD ($T < 100$ K)	Table S3	N/A
Neutron diffraction	RT and $T \leq 60$ K	N/A
Magnetometry	See main text	Section 10
Heat capacity	Figure S2	N/A
Scanning electron microscopy	Figure S1	N/A

TABLE S2: Room-temperature structural parameters and secondary phase weight fractions for both cryptomelane batches studied in this work. The value for KMO-S1 is from combined Rietveld refinement of synchrotron and neutron diffraction data, whereas the value for KMO-S2 comes only from Rietveld refinement of synchrotron data. QPA composition calculated by mass balance between the as-mixed precursor K:Mn ratio (given in Methods) and the Mn_3O_4/Mn_2O_3 weight fractions from quantitative phase analysis, assuming no net loss of K during synthesis and that all K resides in the cryptomelane phase. Carter–Withers composition is the value of x in $K_xMn_8O_{16}$ back-calculated from the incommensurate structural modulation wavevector $\vec{k}_{\text{struc}}$ via the relation $\vec{k}_{\text{struc}} = x/2\,\vec{c^*}$ proposed by Carter and Withers [1]; this modulation was observed only for KMO-S2, so no value can be obtained for KMO-S1.

	**KMO-S1**	**KMO-S2**
**Target composition**	$K_{1.33}Mn_8O_{16}$	$K_{1.33}Mn_8O_{16}$
**Rietveld composition**	$K_{1.448(3)}Mn_8O_{16}$	N/A
**QPA composition**	$K_{1.447(5)}Mn_8O_{16}$	$K_{1.488(2)}Mn_8O_{16}$
**Carter–Withers composition**	N/A	$K_{1.48167(1)}Mn_8O_{16}$
$a = b$ (Å)	9.84942(9)	9.85069(9)
c (Å)	2.862033(17)	2.862296(15)
Mn_2O_3 (wt.%)	0.049(5)	3.16(7)
Mn_3O_4 (wt.%)	2.8(3)	1.99(4)

2. SCANNING ELECTRON MICROSCOPY

Scanning Electron Microscopy (SEM) of cryptomelane powder was performed using an Ultra 55 FESEM Carl Zeiss unit operated at 20 kV and a working distance of around 12 mm. The sample was prepared in air, by sprinkling the brownish-black powder on 1 mm^2 carbon tape stuck on an aluminum stub. The SEM images are presented in Figure S1.

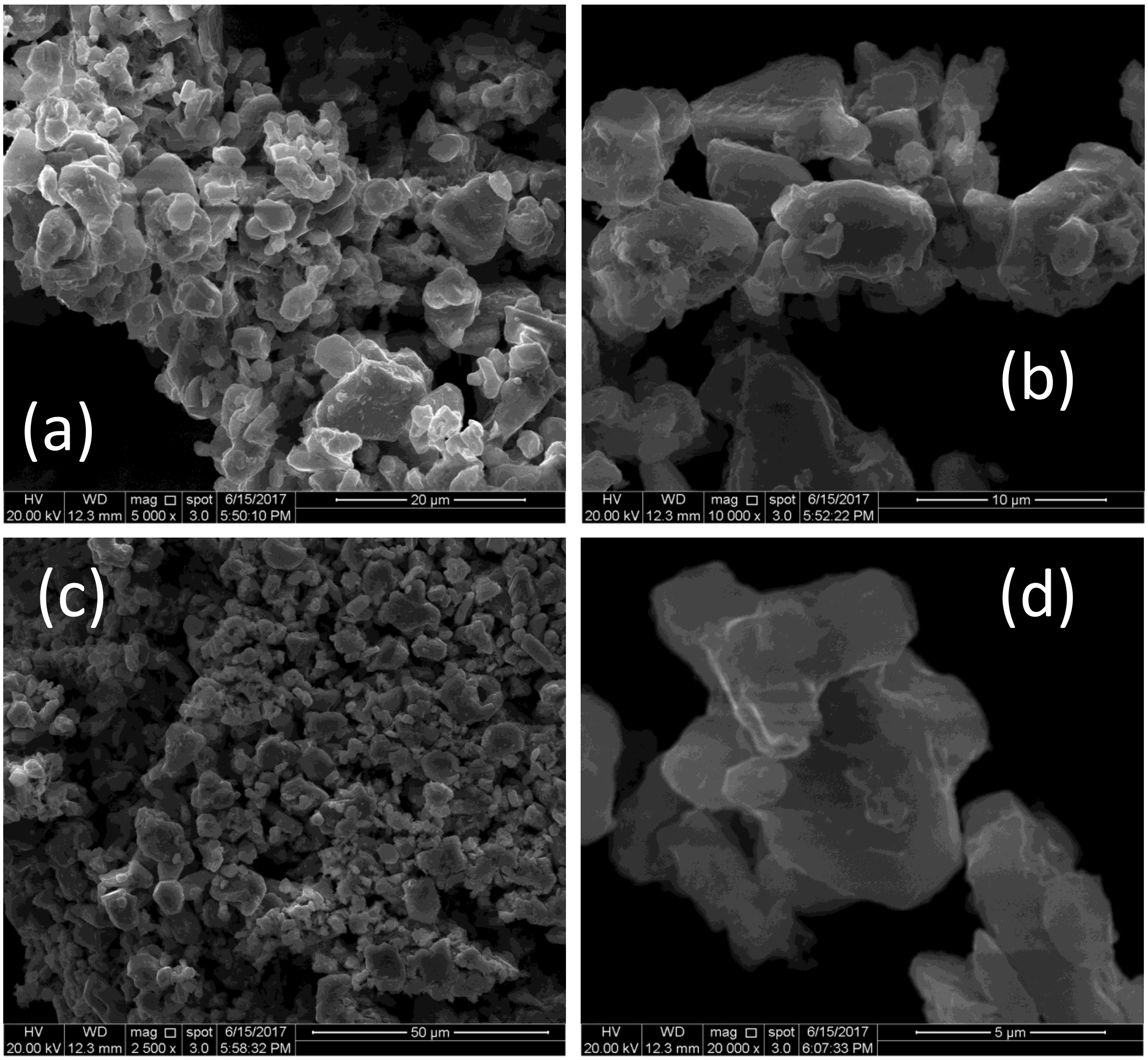

Figure S1: Scanning Electron Microscopy images of cryptomelane (KMO-S1), taken over a range of length-scales.

3. CALCULATING MAGNETIC HEAT CAPACITY

In the manuscript, we present the magnetic heat capacity, C_{mag}. First, the addenda contribution was subtracted, as shown in Figure S2(a). Then the contribution to measured heat capacity from the silver was subtracted, by taking tabulated values for the heat capacity for silver [2], and using the NumPy [3] function INTERP to obtain silver heat capacities at each experimentally-measured temperature. Figure S2(b) shows the silver correction.

After correcting for the silver, the resulting heat capacity data were $C_{\mathrm{tot}} = C_{\mathrm{lat}} + C_{\mathrm{mag}} + C_{\mathrm{electronic}}$. As we saw no sign of a Schottky anomaly, we approximated $C_{\mathrm{electronic}} = 0$. To obtain C_{mag}, we had to remove C_{lat}. We did this using the Debye equation [4]:

$$C_{\mathrm{lat}} = \frac{9nRT^3}{T_{\mathrm{D}}^3} \int_0^{\frac{T_{\mathrm{D}}}{T}} \frac{x^4 e^x}{(e^x - 1)^2} dx \tag{1}$$

which we fit to the experimental heat capacity data (after correcting for the contributions from silver and the addenda) using the SciPy [5] function OPTIMIZE.CURVE_FIT, in the range 60 K to 80 K. Two parameters were optimized in the fit: the Debye temperature T_{D}, and a dimensionless multiplicative scale factor p applied to the whole right-hand side of Equation 1; no additional free offset term was included, so that $C_{\mathrm{lat}} \to 0$ as $T \to 0$ by construction, consistent with the physical requirement on a lattice heat capacity. The value obtained was $T_{\mathrm{D}} = 301.5(11)$ K. Figure S2(c) shows this fit, and Figure S2(d) shows the lattice contribution subtracted to obtain C_{mag}.

Uncertainty in T_{D} and p was obtained from the fit's parameter covariance matrix, and propagated to C_{lat} and C_{mag} using a linearized (delta-method) numerical Jacobian, which correctly accounts for the strong correlation between T_{D} and p.

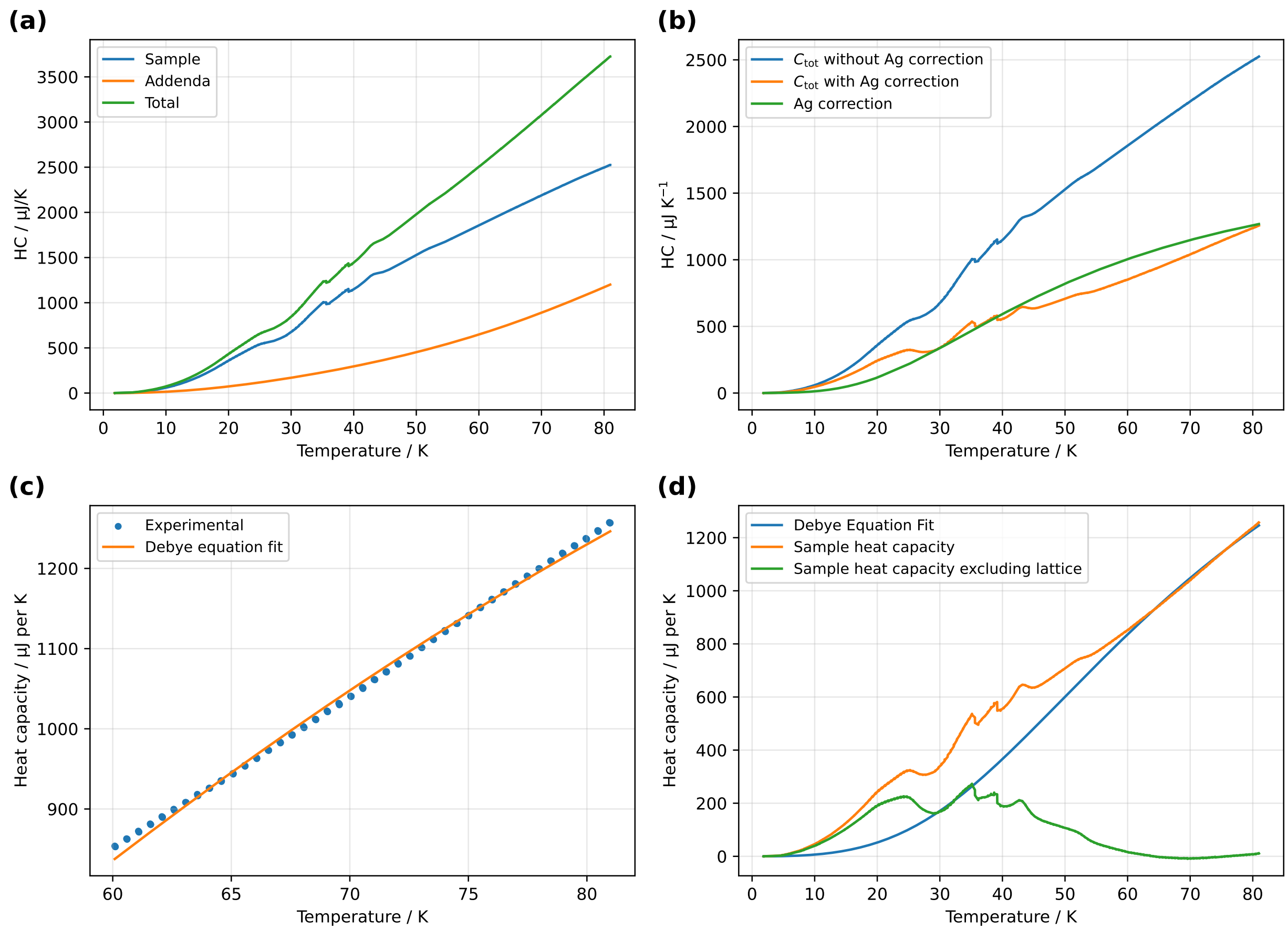

Figure S2: (a) Measured sample heat capacity (KMO-S1), the measured addenda heat capacity, and the sample heat capacity with addenda subtracted. (b) Heat capacity (excluding addenda), the calculated silver contribution, and the heat capacity corrected for both addenda and silver. (c) Fit of Equation 1 to the experimentally-measured heat capacity (corrected for both addenda and silver) between 60 K and 80 K. (d) Raw experimental heat capacity (corrected for addenda and silver), the calculated lattice contribution C_{lat}, and the resulting calculated C_{mag}.

4. LOW-TEMPERATURE POWDER DIFFRACTION

4.1. Neutron powder diffraction

Here we present the full low-temperature neutron diffraction patterns in d-spacing, as obtained from the D20 instrument at Institut Laue-Langevin (λ = 2.4108 Å). Figure S3 shows the raw diffraction patterns for T = 60 K, 47 K, 44 K, 40 K, and 2.5 K. Figure S4 shows the difference patterns X-60 K, where X= 47 K, 44 K, 40 K, and 2.5 K.

Figure S5 shows selected magnetic neutron peak intensities as a function of temperature from the ILL neutron diffraction data measured on D20.

Alongside our neutron diffraction measurements which were made under isothermal conditions after thermalization, we also measured continuously during cooling (λ = 2.4108 Å). Such a dataset is not appropriate for Rietveld refinement, which aims to extract parameters which are often temperature-dependent. However, this dataset is useful simply for ruling out the possibility of temperature dependence of the magnetic propagation vectors $\vec{k}_{\text{mag}}$ within a temperature regime. We present this in Figure S6, where this possibility is clearly ruled out.

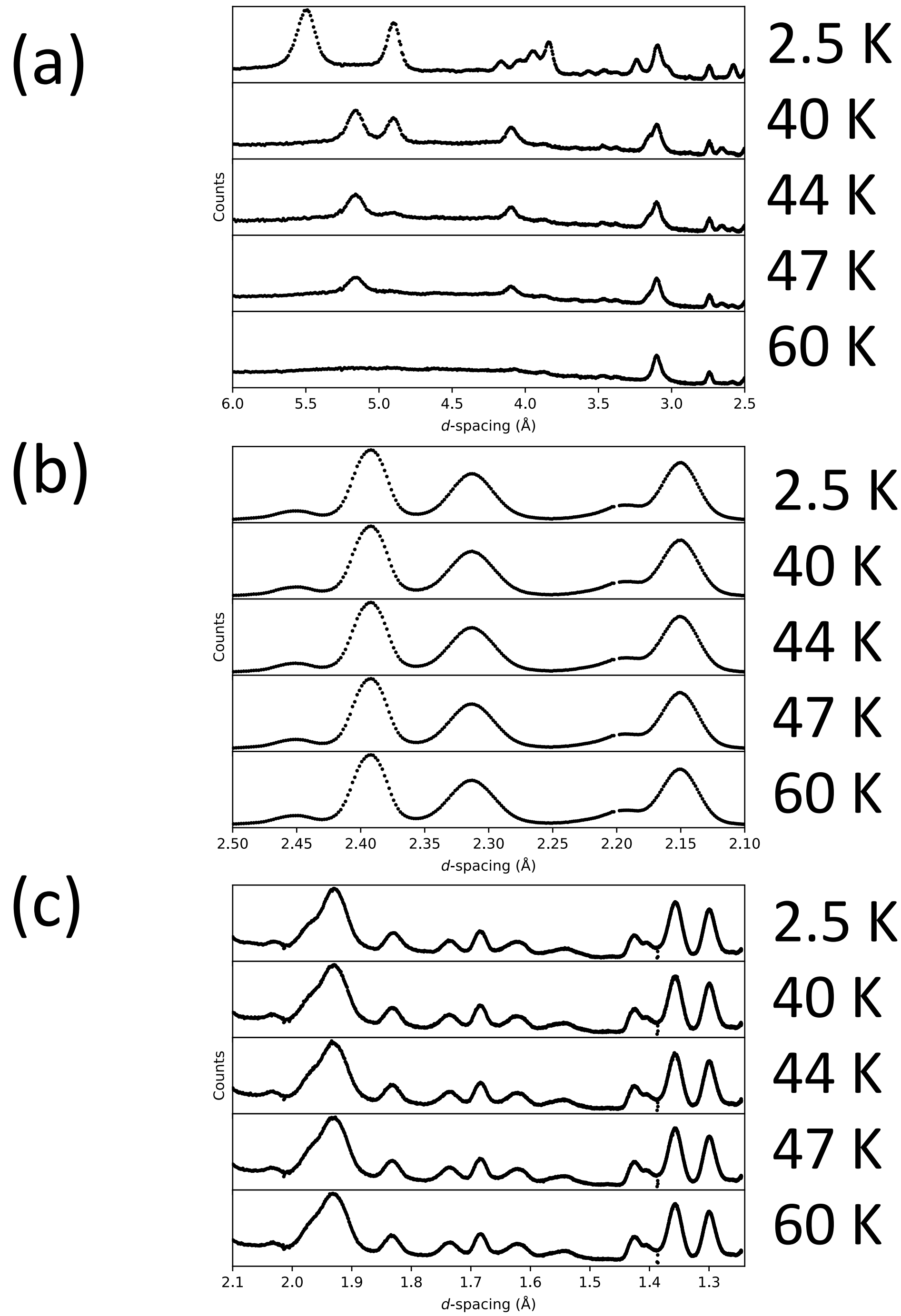

Figure S3: The raw low-temperature neutron diffraction data as obtained for the KMO-S1 sample, in d-spacing, as obtained from the D20 instrument at Institut Laue-Langevin. Temperatures are $T = 60\,\mathrm{K}$, $47\,\mathrm{K}$, $44\,\mathrm{K}$, $40\,\mathrm{K}$, and $2.5\,\mathrm{K}$. (a), (b), and (c) are at different y-scales.

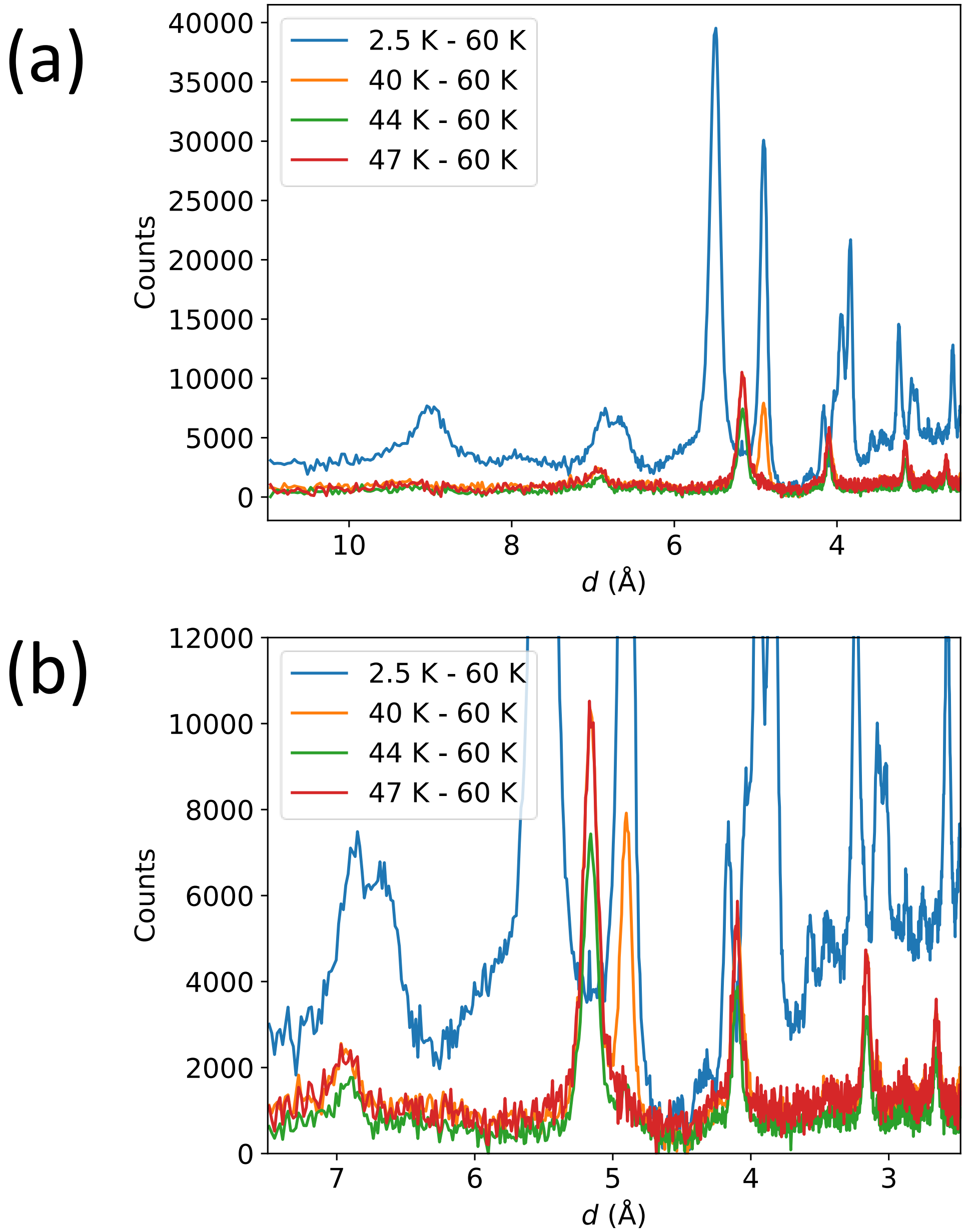

Figure S4: The low-temperature neutron diffraction difference patterns X-60 K (KMO-S1), where X= 47 K, 44 K, 40 K, and 2.5 K, in d-spacing. (a) shows the full d-range, whereas (b) is limited to the d- and y- range of the peaks for $T_3 < T < T_2$. From the D20 instrument at Institut Laue-Langevin.

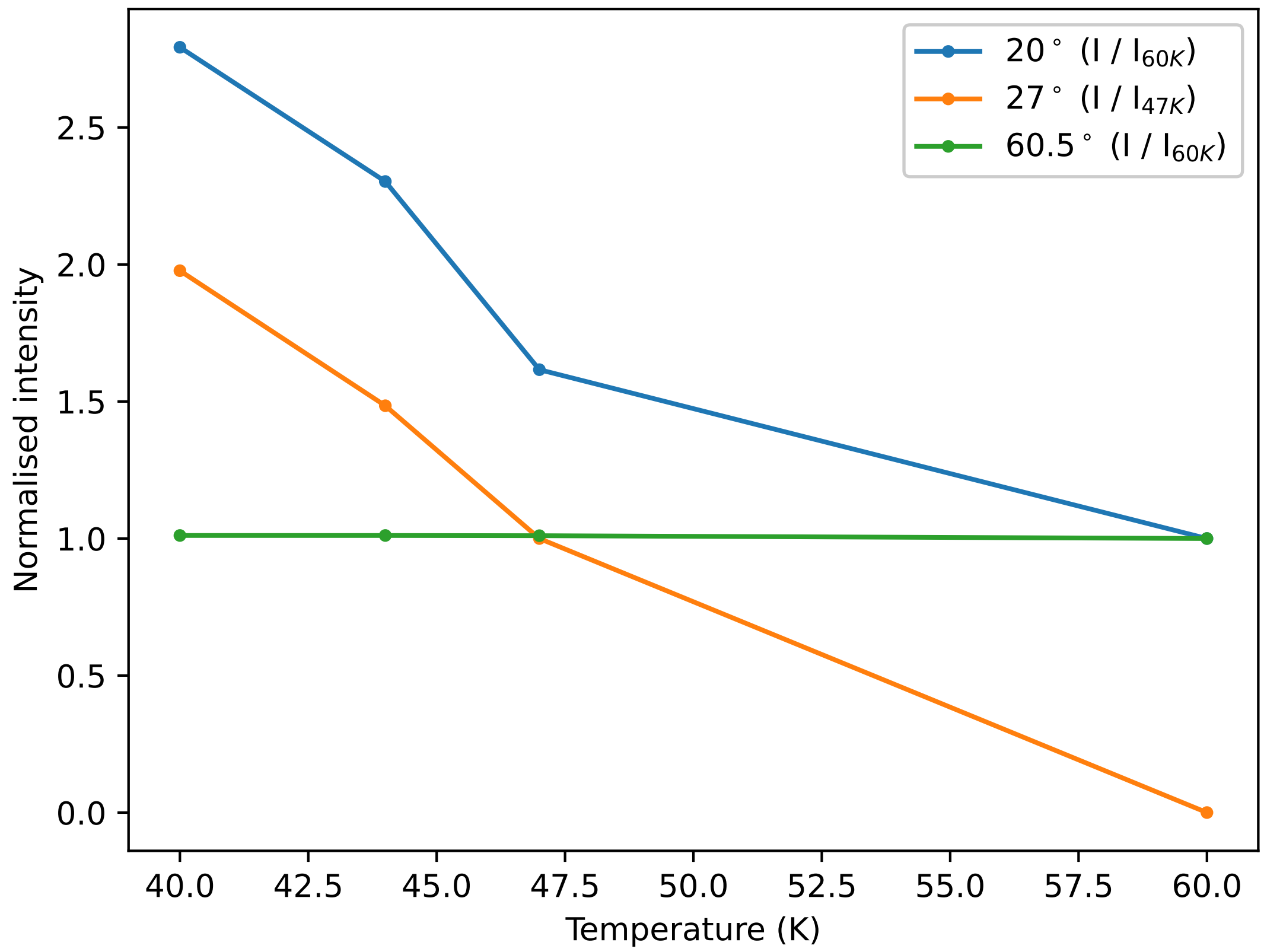

Figure S5: Normalized intensities for the $\vec{k}_{\mathrm{mag}} = (0, 0, 0)$ peak at 20° (the [1,1,0] peak in tetragonal notation), with both a nuclear and magnetic contribution, a $\vec{k}_{\mathrm{mag}} = (0, 0, \sim 0.37)$ peak at 27° with purely magnetic contribution (the [0,0,∼0.55] peak in tetragonal notation), and a nuclear Bragg peak with no magnetic contribution at 60.5° (the [2,1,1] peak). Intensities were obtained by fitting a Gaussian peakshape to the experimental neutron diffraction data obtained from D20 at the ILL, for the KMO-S1 sample.

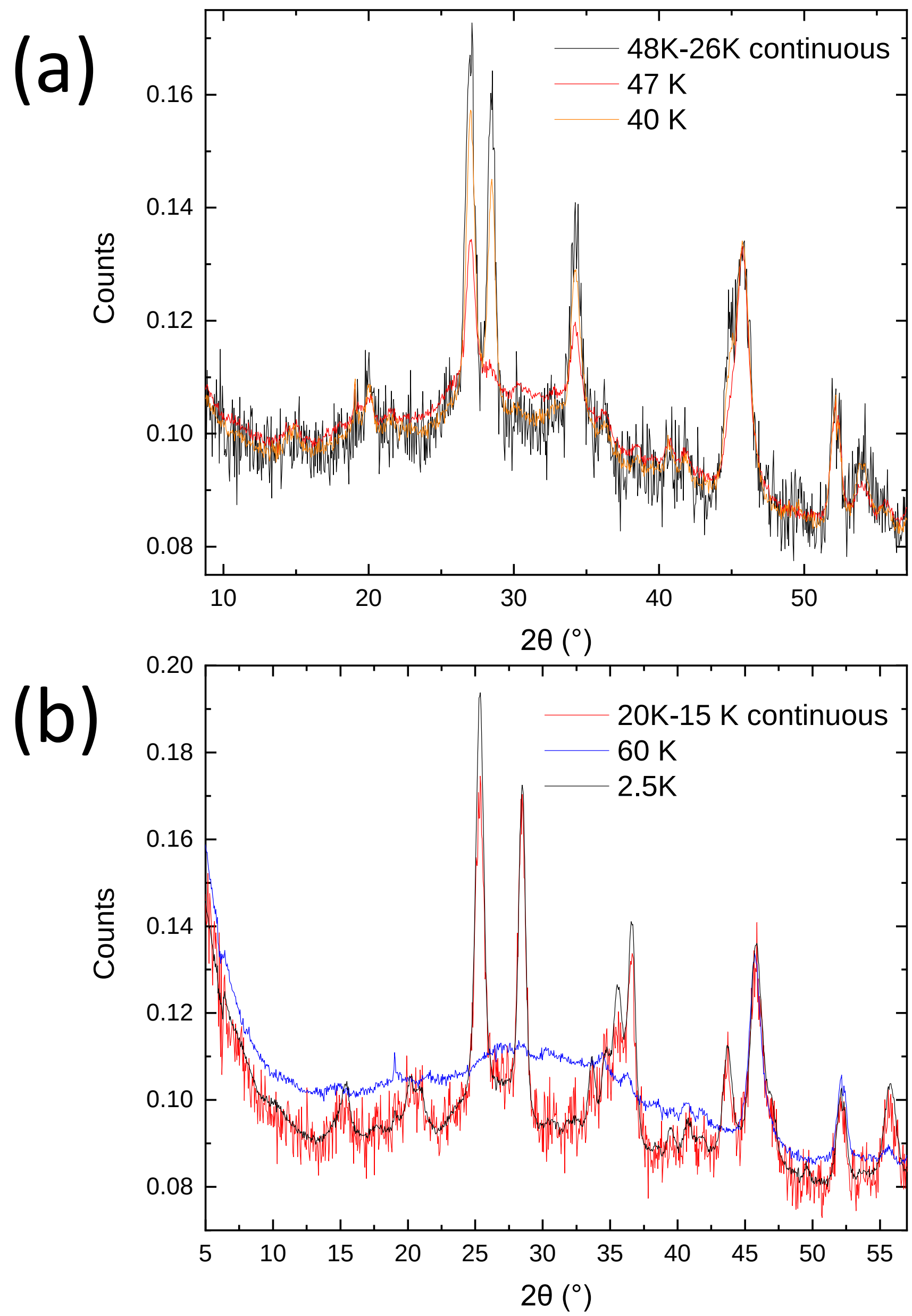

Figure S6: A comparison of the isothermal and non-isothermal neutron diffraction patterns over some temperature ranges, measured on KMO-S1 at the D20 instrument, ILL. (a) shows the $T_3 < T < T_2$ temperature regime, with the only differences between isothermal and non-isothermal data clearly attributable to magnetic ordering of Mn_3O_4 between 47 K and 40 K. (b) shows that all the peaks present at 2.5 K have the same position between 20 K and 15 K.

4.2. Laboratory X-ray diffraction

To test for the possibility of structural transitions in cryptomelane in the vicinity of the magnetic transitions, we performed low-temperature X-ray diffraction (XRD) measurements using a Bruker D8 Discover diffractometer ($\lambda = 1.541$ Å), with an Oxford Cryosystems PheniX stage. We find that there are no structural transitions observable with the resolution of the instrument, noting the instrumental resolution is inferior to the synchrotron measurements which observed the structural transition at T_1. Figure S7 shows the low-temperature X-ray diffraction data. As discussed elsewhere, there is in reality a subtle monoclinic distortion to this tetragonal space group, but this is not resolvable using the low resolution of the laboratory diffractometer, and so Figure S8 shows an example Rietveld refinement and the temperature dependence of the lattice parameters a and c using the $I4/m$ space group. Table S3 lists the refined parameters.

Several peaks in these diffraction patterns arise from the low-temperature sample-holder, and have been excluded from refinements. These are the peaks at 43.6 ° ($d = 2.07$ Å), 44.8 ° ($d = 2.02$ Å), 50.8 ° ($d = 1.79$ Å), and 52.2 ° ($d = 1.75$ Å).

No preferred orientation function was used in the refinement of these diffraction patterns.

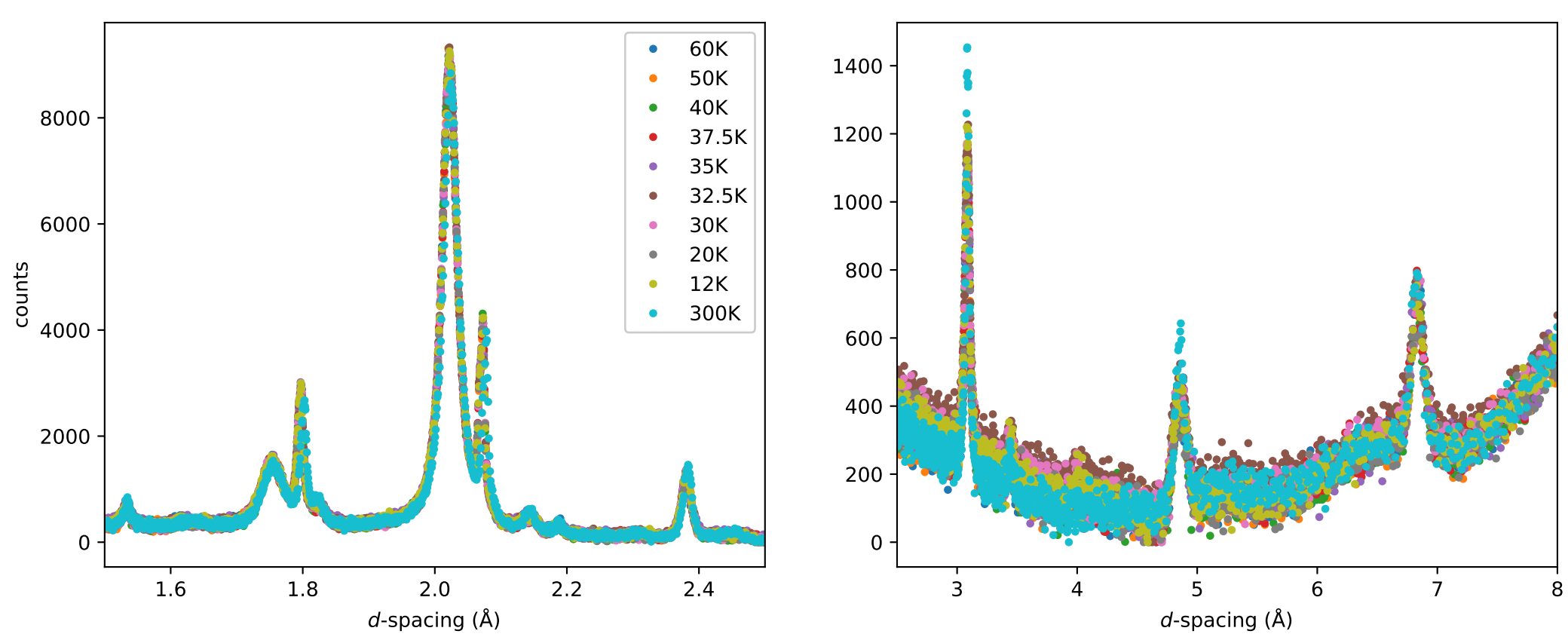

Figure S7: The raw laboratory X-ray diffraction data as obtained for the KMO-S1 sample. Data are presented between 1.5 Å and 2.5 Å (left) and 2.5 Å and 8 Å (right), in d-spacing. Temperatures are (in order of measurement) 60 K, 50 K, 40 K, 37.5 K, 35 K, 32.5 K, 30 K, 20 K, 12 K, and 300 K after subsequent heating.

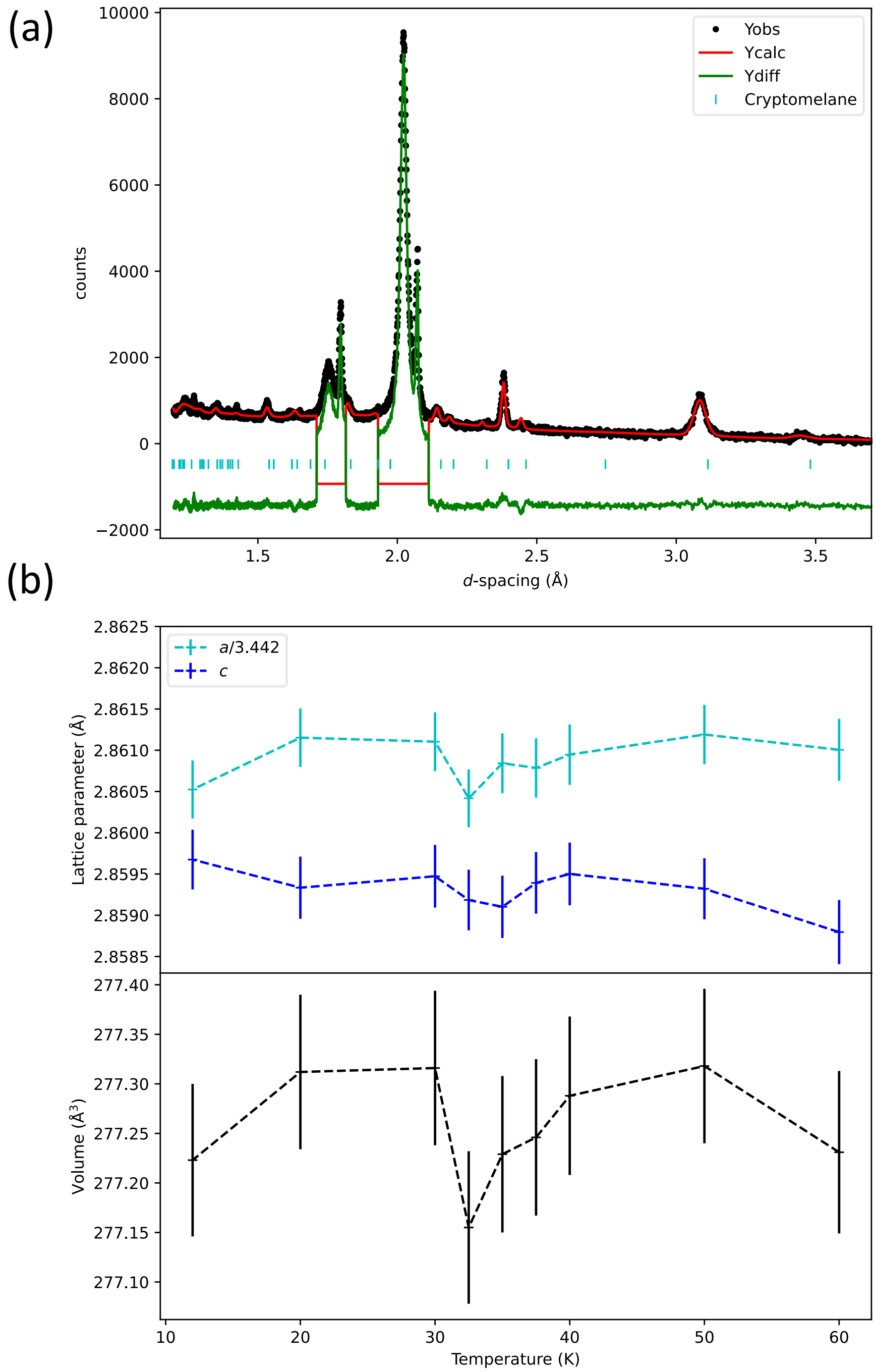

Figure S8: (a) shows an example Rietveld refinement, of diffraction data obtained using low-temperature laboratory X-ray diffraction at 12 K, for the KMO-S1 sample. Several peaks in these diffraction patterns are due to the low-temperature sample-holder, and have been excluded from refinements. (b) shows the temperature dependence of the lattice parameters a (represented as $a/3.442$ for scaling), c, and unit cell volume V at low temperatures from these X-ray Rietveld refinements.

T (K)	a (Å)	c (Å)	V ($Å^3$)	R_{wp}	R_p	χ^2
60 (↓)	9.8476(13)	2.8588(4)	277.23(8)	3.83	2.96	2.05
50 (↓)	9.8482(12)	2.8593(3)	277.32(8)	3.74	2.88	1.97
40 (↓)	9.8474(13)	2.8595(4)	277.29(8)	3.80	2.91	2.04
37.5 (↓)	9.8468(12)	2.8594(4)	277.25(8)	3.81	2.94	2.06
35 (↓)	9.8470(13)	2.8591(4)	277.23(8)	3.84	2.97	2.09
32.5 (↓)	9.8456(12)	2.8592(4)	277.16(8)	3.77	2.94	2.01
30 (↓)	9.8479(12)	2.8595(4)	277.32(8)	3.86	3.00	2.12
20 (↓)	9.8481(12)	2.8593(4)	277.31(8)	3.82	2.96	2.09
12 (↓)	9.8460(12)	2.8597(4)	277.23(8)	3.79	2.91	2.05
300 (↑)	9.8512(9)	2.8624(3)	277.78(6)	3.80	2.89	2.05

TABLE S3: Lattice parameters of $I4/m$ cryptomelane KMO-S1 obtained by Rietveld refinement of the variable-temperature laboratory X-ray diffraction. Uncertainties are shown in brackets. Site occupancy was fixed at 1 for Mn and O, and 1/3 for K. The arrow next to the temperature indicates whether the data were collected on warming or cooling of the sample. R_{wp}, R_p, and χ^2 are each rounded to 3 significant figures. We note that a tetragonal $I4/m$ space group is used here as the monoclinic distortion is not resolvable using this low-resolution diffraction data.

4.3. Low-temperature synchrotron X-ray diffraction

4.3.1. Introduction

We performed synchrotron X-ray diffraction on a sample of cryptomelane, denoted KMO-S2, between 300 K and 100 K during cooling, in order to probe the behavior through the T_1 transition, Figure S9 and Figure S10.

KMO-S2 was synthesized following the same technique and target composition as KMO-S1 (see Section 1), but combined neutron and X-ray diffraction were not possible for KMO-S2, so we cannot claim an independently refined composition for it. We suspect it is similar, given the near-identical lattice parameters [Table S2], but it cannot be identical due to slightly different impurity phase fractions and value of T_1 temperature.

KMO-S2 features the same Mn_3O_4 (space group $I4_1/amd$) and Mn_2O_3 (space group $Ia\bar{3}$; modelled with $Pbca$ at low temperatures) impurities as KMO-S1, the sample used for magnetic neutron diffraction.

During the synchrotron measurements on the I11 instrument at Diamond Light Source, $\lambda = 0.825223$ Å and measurements were performed for 20 seconds at discrete 1 K intervals.

We found that, in the vicinity of T_1, several new peaks emerged at 6.45°, 14.05°, $\sim 15.65°$ (a shoulder), and 18.40° (corresponding to $d = 7.35$ Å, 3.37 Å, 3.03 Å, and 2.58 Å, respectively), as shown in Figure S9. These new peaks are associated with an incommensurate non-magnetic structural modulation, as X-ray diffraction is insensitive to unpaired spins. As described in the main text, these peaks can be indexed to $\vec{k}_{\text{struc}} = (0, 0, k_z)$, via assignment of $(0, \bar{1}, 1 - k_z)$ ($d = 7.35$ Å), $(\bar{1}, 0, k_z)$ ($d = 3.37$ Å), $(0, 0, k_z)$ ($d = 3.03$ Å), and $(\bar{2}, 0, k_z)$ ($d = 2.58$ Å). The value of k_z is determined in Section 4.3.4 for KMO-S2. It should be noted that KMO-S2 likely does not have exactly the same K content as KMO-S1, as evidenced by the differing temperature of T_1 and by the differing secondary-phase weight fractions of the two batches (Table S2); their closely comparable room-temperature lattice parameters (also Table S2) nonetheless indicate the two batches are compositionally similar. Consequently, the exact value of k_z may differ slightly between KMO-S1 and KMO-S2.

We tabulate the crystallographic parameters of monoclinic cryptomelane at 100 K as obtained from Rietveld refinement of the synchrotron X-ray diffraction data in Table S4.

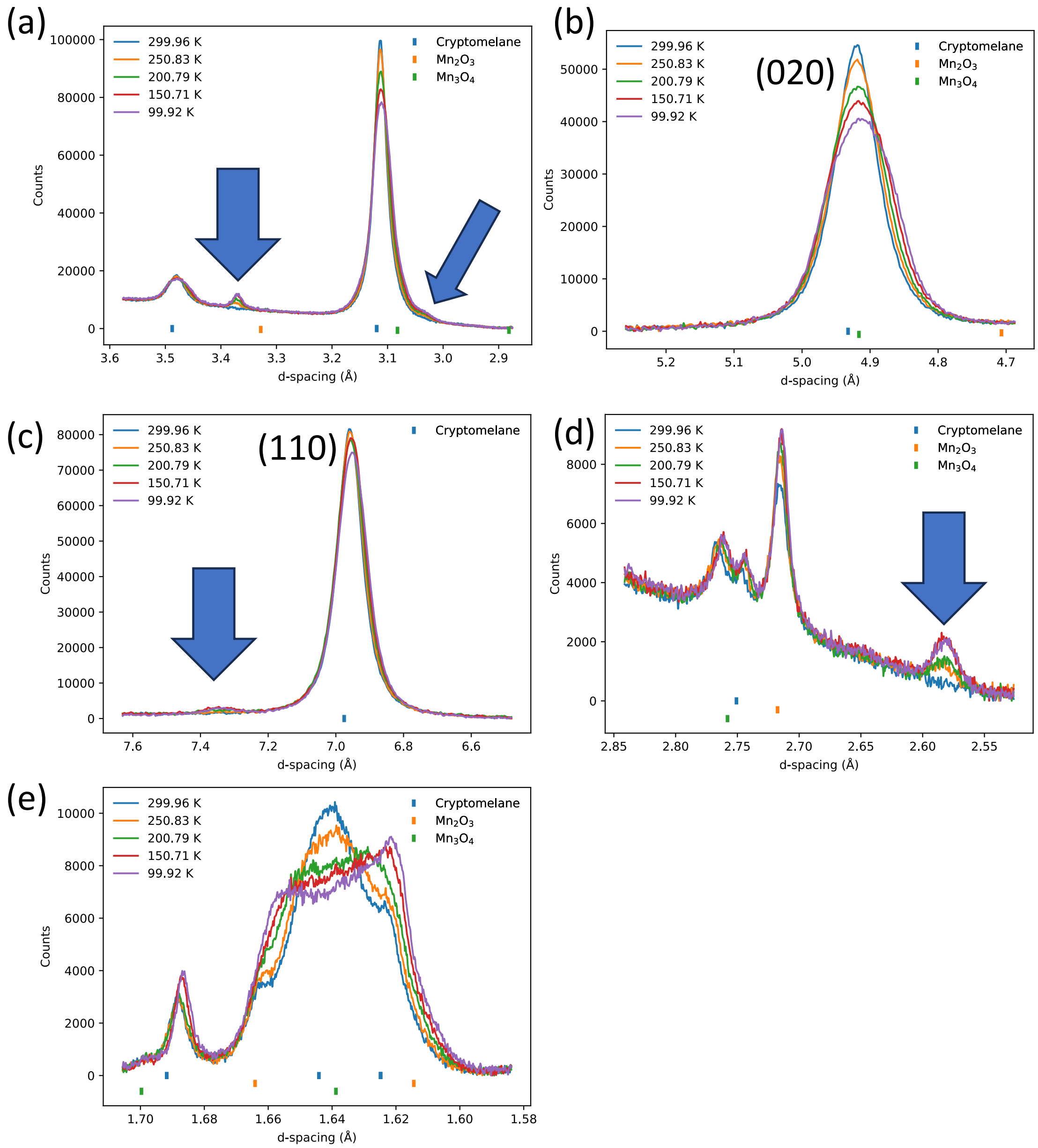

Figure S9: Various changes in the diffraction pattern of KMO-S2 on cooling from 300 K to 100 K. (a) shows the emergence of an incommensurate non-magnetic peak around $d = 3.37$ Å and another at 3.03 Å. (b) shows broadening of the (020) peak due to the growing monoclinic distortion. (c) shows the emergence of an incommensurate non-magnetic peak around $d = 7.35$ Å. (d) shows the emergence of an incommensurate non-magnetic peak around $d = 2.58$ Å. (e) shows splitting of the (060) tetragonal peak into two peaks as the unit cell becomes increasingly monoclinic, overlapping with impurity peaks. In all plots, tickmarks due to tetragonal cryptomelane ($I4/m$) and the two known impurities Mn_2O_3 and Mn_3O_4 are also shown.

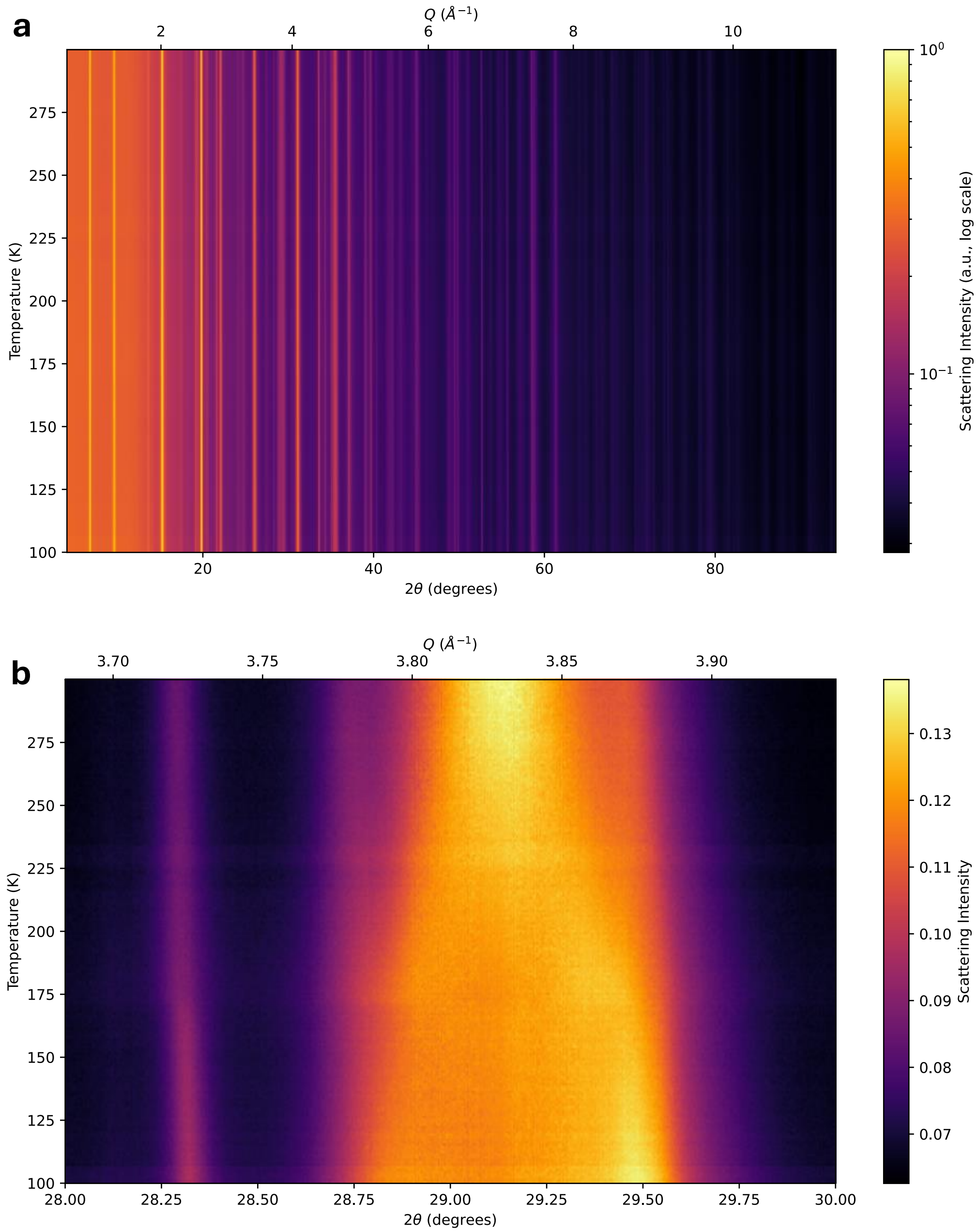

Figure S10: Heat map of the diffraction pattern of KMO-S2 on cooling from 300 K to 100 K. (a) shows the full data, with the intensities represented by a logarithmic color scale. (b) shows very subtle broadening of the (020) peak due to the monoclinic distortion.

TABLE S4: Crystallographic parameters of cryptomelane at 100 K, as obtained from Rietveld refinement of synchrotron X-ray diffraction data on KMO-S2 from I11 assuming monoclinic symmetry. Atomic displacement parameters are refined but fixed equal for same-element sites. χ^2, R_{wp}, and R_{exp} are rounded to 3 significant figures. Refinement was carried out in the conventional $C2/m$ setting (unique axis b); all parameters are presented here transformed into the equivalent $A2/m$ setting (unique axis c) so that the tunnel-parallel axis is consistently c, as in the $I4/m$ parent cell.

Space group	$A2/m$
a / Å	9.8437(5)
b / Å	13.9123(6)
c / Å	2.85768(2)
γ / °	135.0149(18)
V / Å^3	276.66(2)
χ^2	11.834
R_{wp}	2.74
R_{exp}	0.797
K1 at $4g$ (0, 0, z)	
z(K1)	0.3732(8)
occupancy(K1)	0.3533(8)
B_{eq}(K1) / Å^2	0.60(9)
Mn1 at $4i$ (x, y, 0)	
x(Mn1)	0.5191(4)
y(Mn1)	0.1669(3)
B_{eq}(Mn1) / Å^2	0.152(10)
Mn2 at $4i$ (x, y, 0)	
x(Mn2)	0.1812(3)
y(Mn2)	0.3495(3)
B_{eq}(Mn2) / Å^2	0.152(10)
O1 at $4i$ (x, y, 0)	
x(O1)	0.3352(9)
y(O1)	0.1919(6)
B_{eq}(O1) / Å^2	0.06(3)
O2 at $4i$ (x, y, 0)	
x(O2)	-0.0527(12)
y(O2)	0.1571(9)
B_{eq}(O2) / Å^2	0.06(3)
O3 at $4i$ (x, y, 0)	
x(O3)	0.6941(9)
y(O3)	0.1495(5)
B_{eq}(O3) / Å^2	0.06(3)
O4 at $4i$ (x, y, 0)	
x(O4)	0.3592(10)
y(O4)	0.5428(8)
B_{eq}(O4) / Å^2	0.06(3)

4.3.2. Sequential refinement of synchrotron data: monoclinic cell

We also see in Figure S9 that the monoclinic distortion becomes more significant on cooling. While at 300 K, it is possible to fit the data using the tetragonal $I4/m$ space group (as we do in the main text), on cooling to 100 K the data cannot be fit satisfactorily with this cell, and a monoclinic cell is needed. We attribute this to a monoclinic→tetragonal transition at T_1. To study this further, we perform sequential Rietveld refinement using a monoclinic $A2/m$ unit cell, with example fits shown in Figure S11. In this refinement, K occupancy is fixed at 0.35334 based on a preliminary Rietveld refinement of the 100 K synchrotron data using the $A2/m$ unit cell, though we note this is not refined in conjunction with neutron data so is likely less reliable than the value quoted for KMO-S1 in Table S2.

The results of the sequential Rietveld refinement are shown in a series of following figures. Figure S12 shows the dependence of lattice parameters of the monoclinic unit cell with temperature. Figure S13 shows the dependence of lattice parameters on temperature, expressed as pseudo-tetragonal parameters as defined in the figure, and Figure S14 shows this same dataset but as a percentage increase from its starting value. Figure S15 shows the dependence of refined unit cell volume on temperature. Figure S16 shows the dependence of the Stephens' parameters [6] used to model anisotropic peak broadening. Figure S17 shows the temperature dependence of the atomic displacement parameters (ADPs) for each atomic site. Figure S18 and Figure S19 show the shift in Mn and K positions with temperature, respectively. Figure S20 shows the dependence of Mn-O bond lengths on temperature for the two unique Mn sites in the unit cell, and Figure S21 shows the MnO_6 octahedral distortion (bond length distortion index and bond angle variance) as a function of temperature. Figure S22 shows the equatorial O-O distances for both MnO_6 octahedra, as a function of temperature. Figure S23 shows the bond valence sum of the two Mn sites, assuming $R_0 = 1.762$ Å and $B_0 = 0.34$. Finally, Figure S24 shows the R_{wp} as a function of temperature in this sequential refinement.

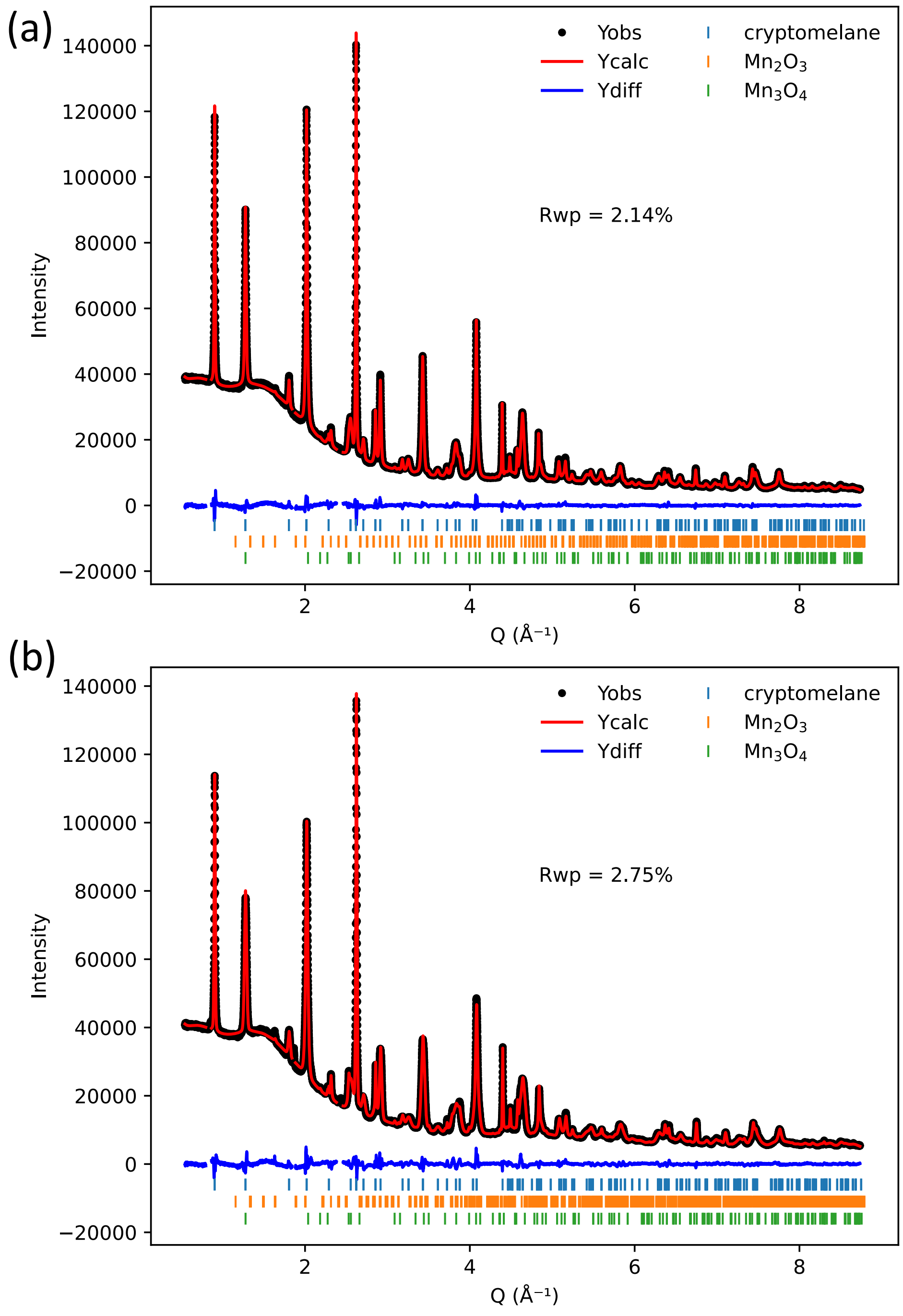

Figure S11: Example Rietveld fits using the monoclinic $A2/m$ space group, from a sequential Rietveld refinement on KMO-S2, at (a) 300 K and (b) 100 K. Data were collected on cooling. Data in the vicinity of incommensurate non-magnetic peaks are excluded from the fit and do not contribute to R_{wp}.

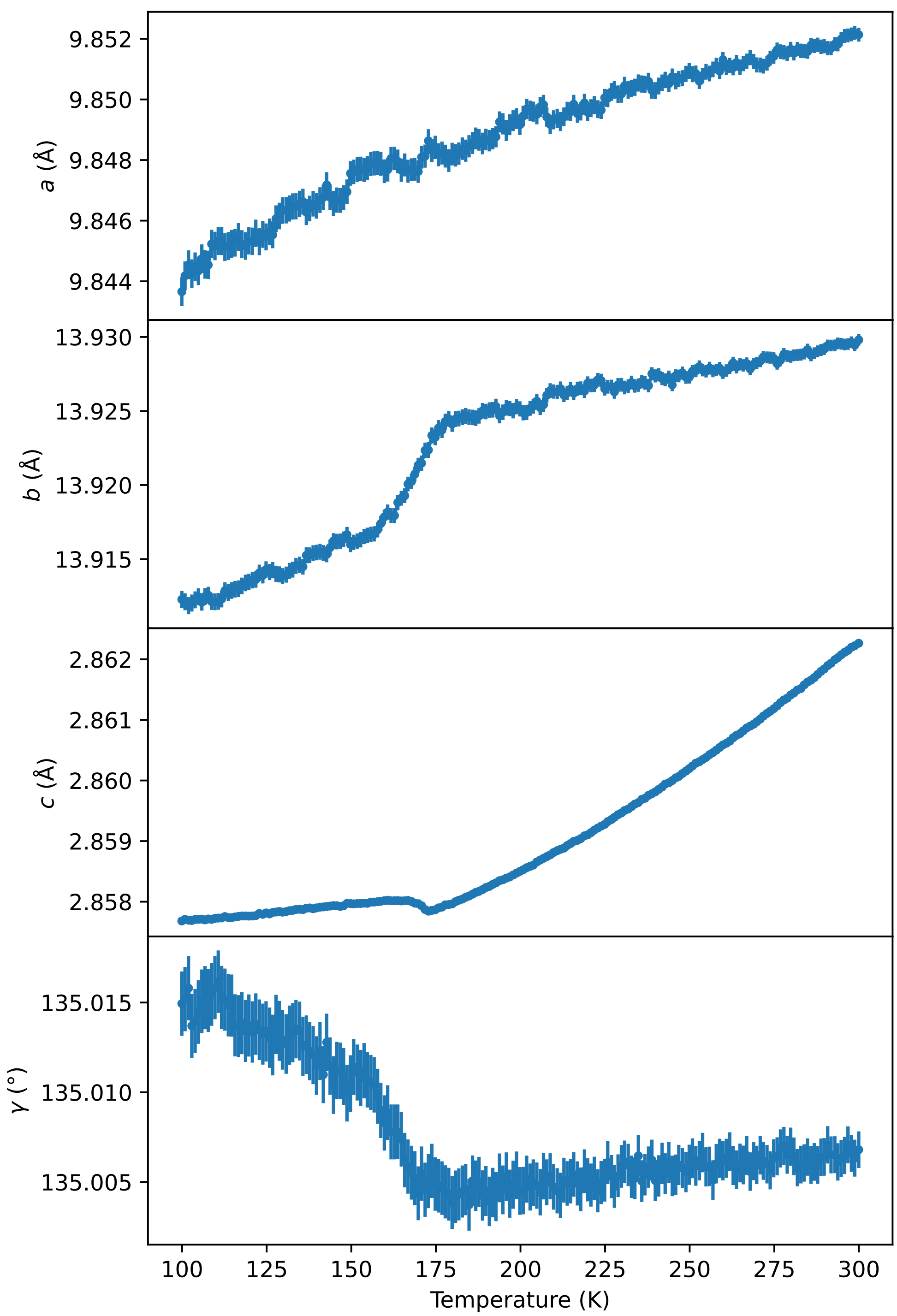

Figure S12: Refined unit cell parameters against temperature for monoclinic cryptomelane (KMO-S2) between 100 K and 300 K, as obtained by sequential Rietveld refinement of diffraction data, where diffraction patterns are analyzed in ascending order of temperature. Cell parameters (a, b, c, γ) are shown in the $A2/m$ setting (unique axis c, parallel to the nanotunnels); refinement itself was performed in the conventional $C2/m$ setting.

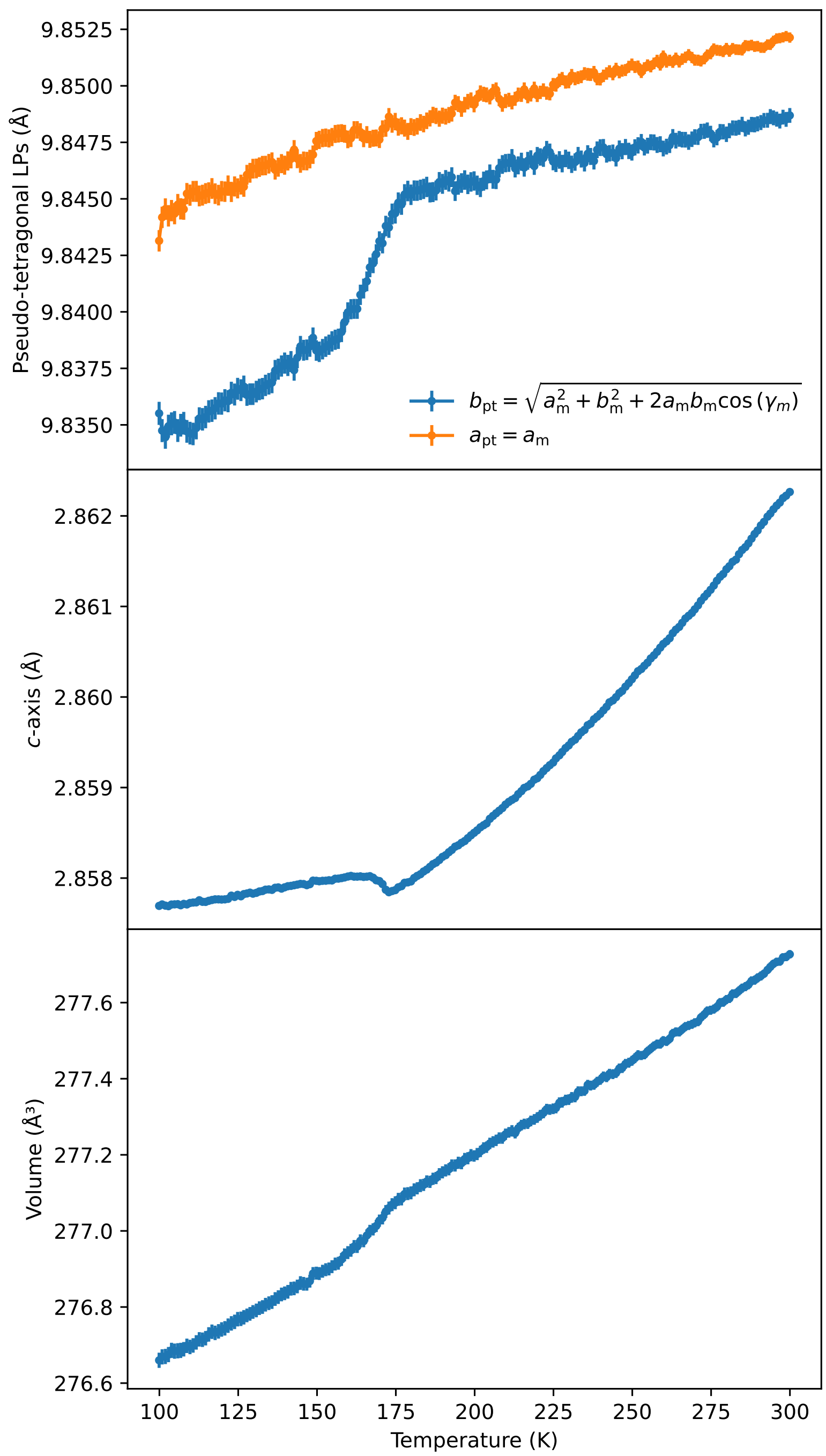

Figure S13: Refined pseudo-tetragonal unit cell parameters against temperature for monoclinic cryptomelane between 100 K and 300 K, as obtained by sequential Rietveld refinement of diffraction data (measured on KMO-S2), where diffraction patterns are analyzed in ascending order of temperature. Lower panel is the c-axis (parallel to the nanotunnels), and upper panel are pseudo-tetragonal lattice parameters a_{pt} and b_{pt} of the monoclinic cell (Eqs. 3 and 4 of the main text), defined in the legend.

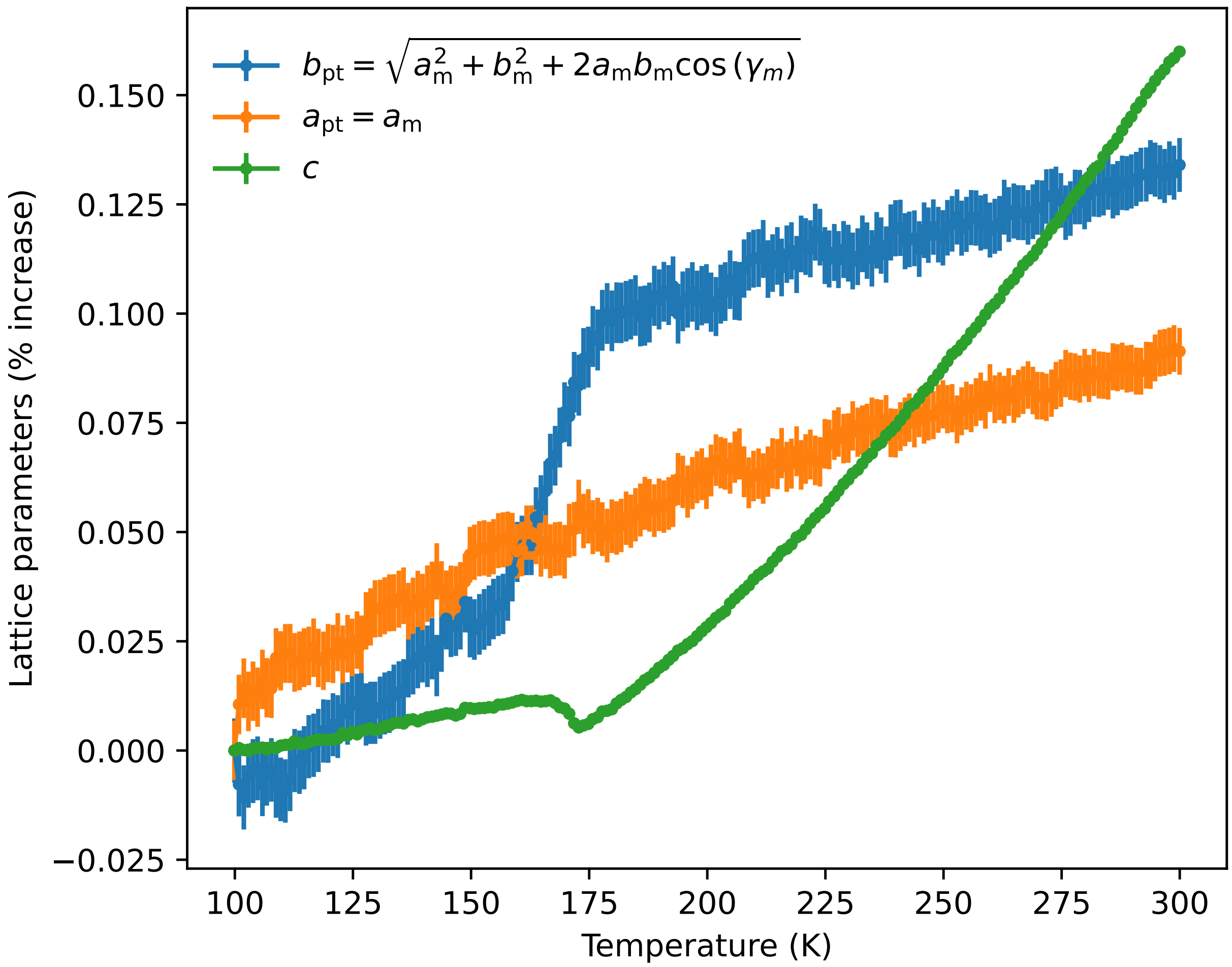

Figure S14: Refined relative unit cell parameters against temperature for monoclinic cryptomelane (KMO-S2) between 100 K and 300 K, as obtained by sequential Rietveld refinement of diffraction data, where diffraction patterns are analyzed in ascending order of temperature. Lower panel is the c-axis (parallel to the nanotunnels), and upper panel are pseudo-tetragonal lattice parameters a_{pt} and b_{pt} of the monoclinic cell, defined in the legend.

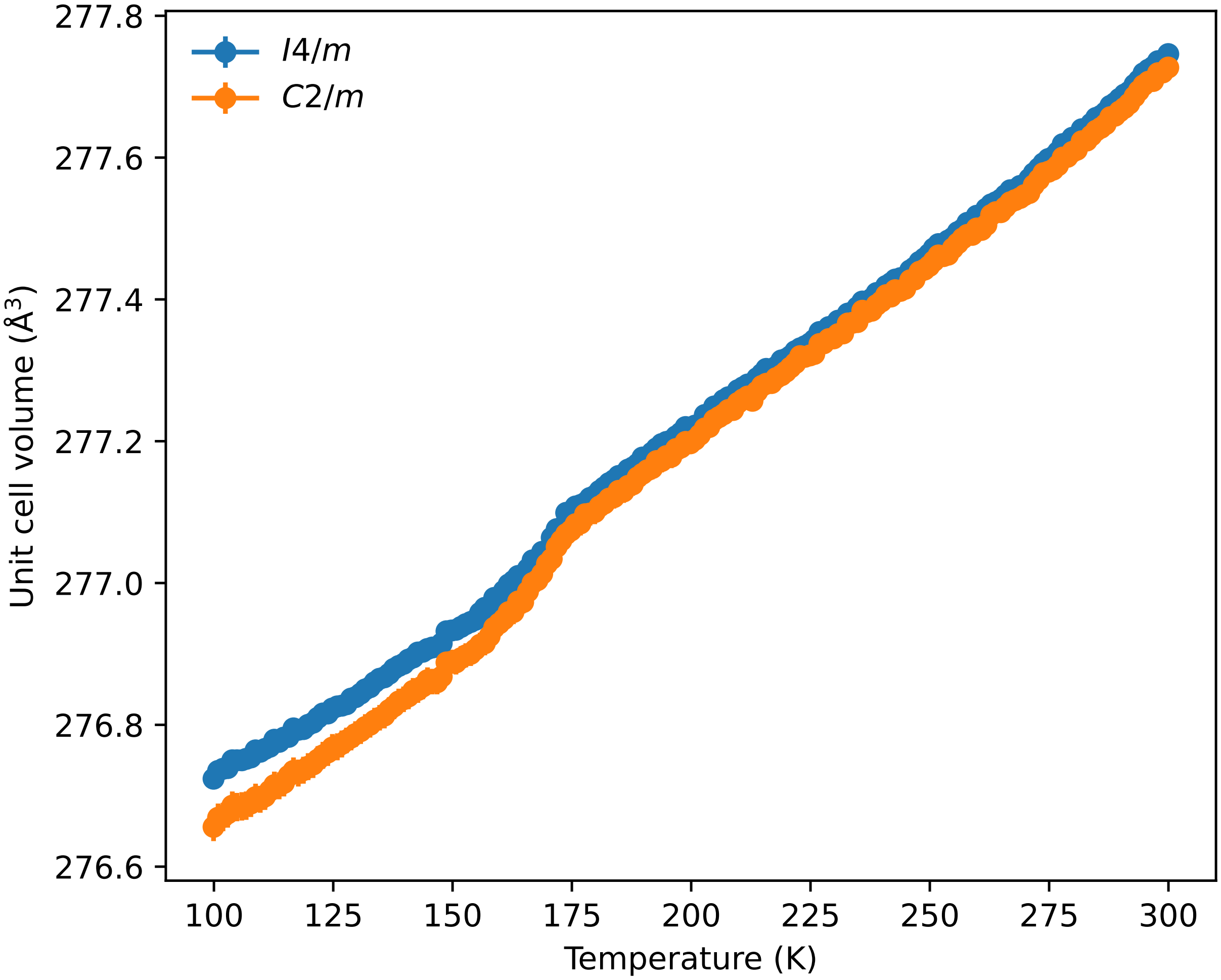

Figure S15: Refined unit cell volume against temperature for cryptomelane (KMO-S2) between 100 K and 300 K, as obtained by sequential Rietveld refinement of diffraction data. The monoclinic (which was refined in $C2/m$ but we present using the alternate $A2/m$ setting elsewhere) unit cell and tetragonal ($I4/m$) unit cell are compared. In both cases, diffraction patterns are analyzed in ascending order of temperature. Error bars are shown for each data point.

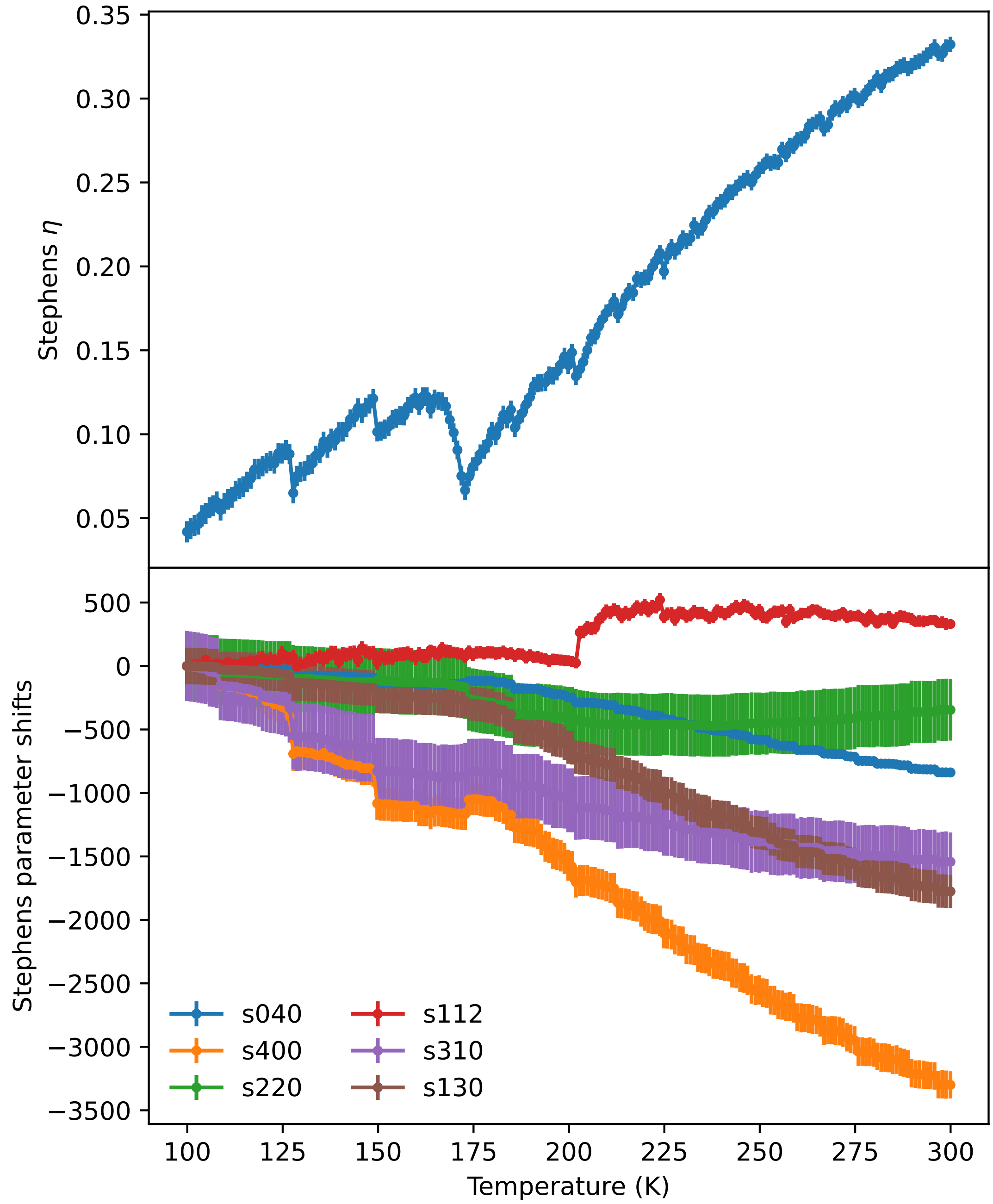

Figure S16: Dependence of Stephens' parameters [6] used to model anisotropic peak broadening, as obtained by sequential Rietveld refinement of variable-temperature diffraction data on cryptomelane (KMO-S2) between 100 K and 300 K. Upper panel is the η parameter. Lower panel shows the relative trend of Stephens' parameters from their value at 100 K where the anisotropic peak broadening is greatest. In the sequential Rietveld refinement that obtained these results, diffraction patterns are analyzed in ascending order of temperature.

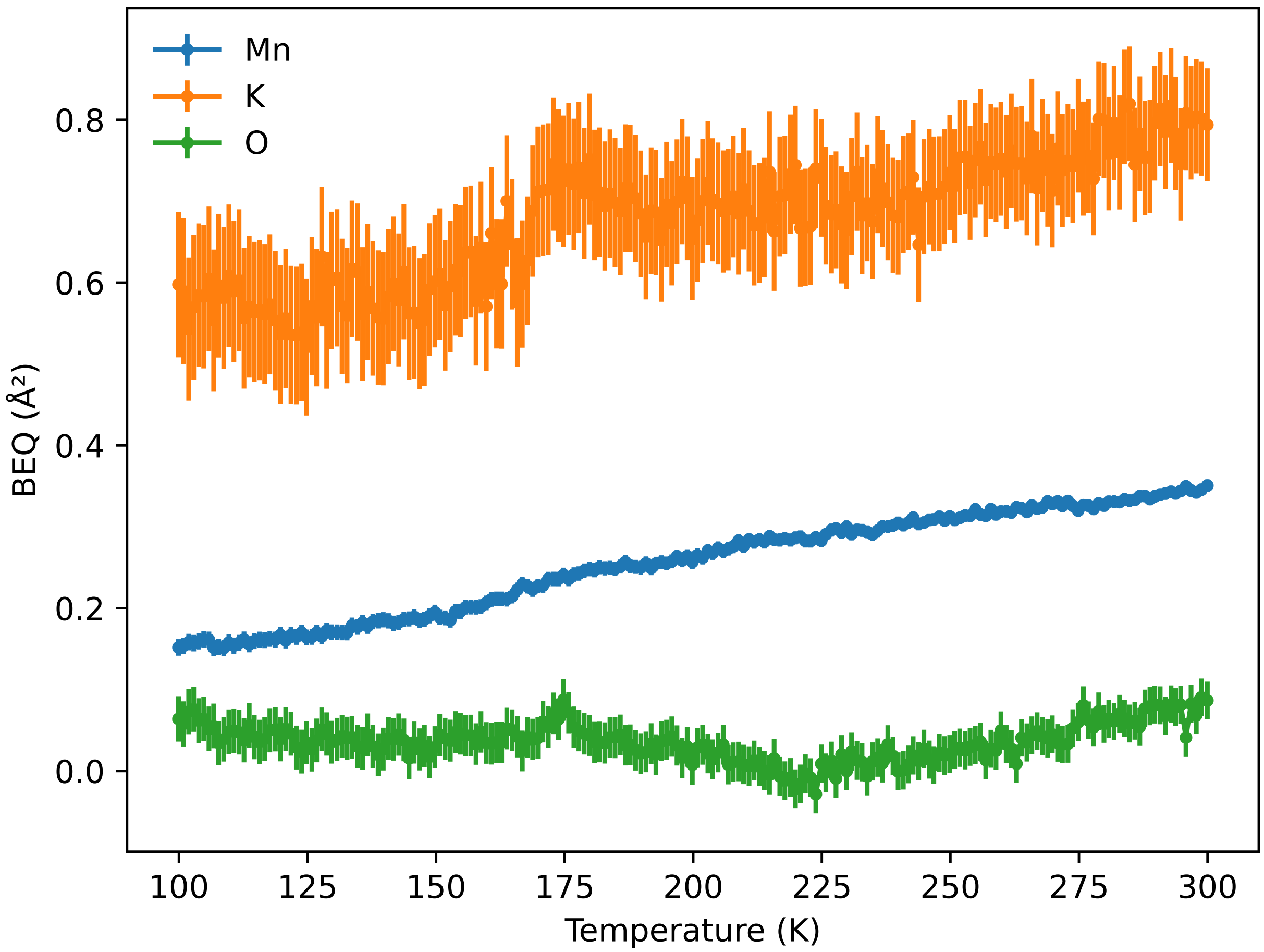

Figure S17: Dependence of atomic displacement parameters for each site, as obtained by sequential Rietveld refinement of variable-temperature diffraction data on cryptomelane (KMO-S2) between 100 K and 300 K. This sequential refinement assumes a monoclinic ($A2/m$) unit cell. Here, diffraction patterns are analyzed in ascending order of temperature.

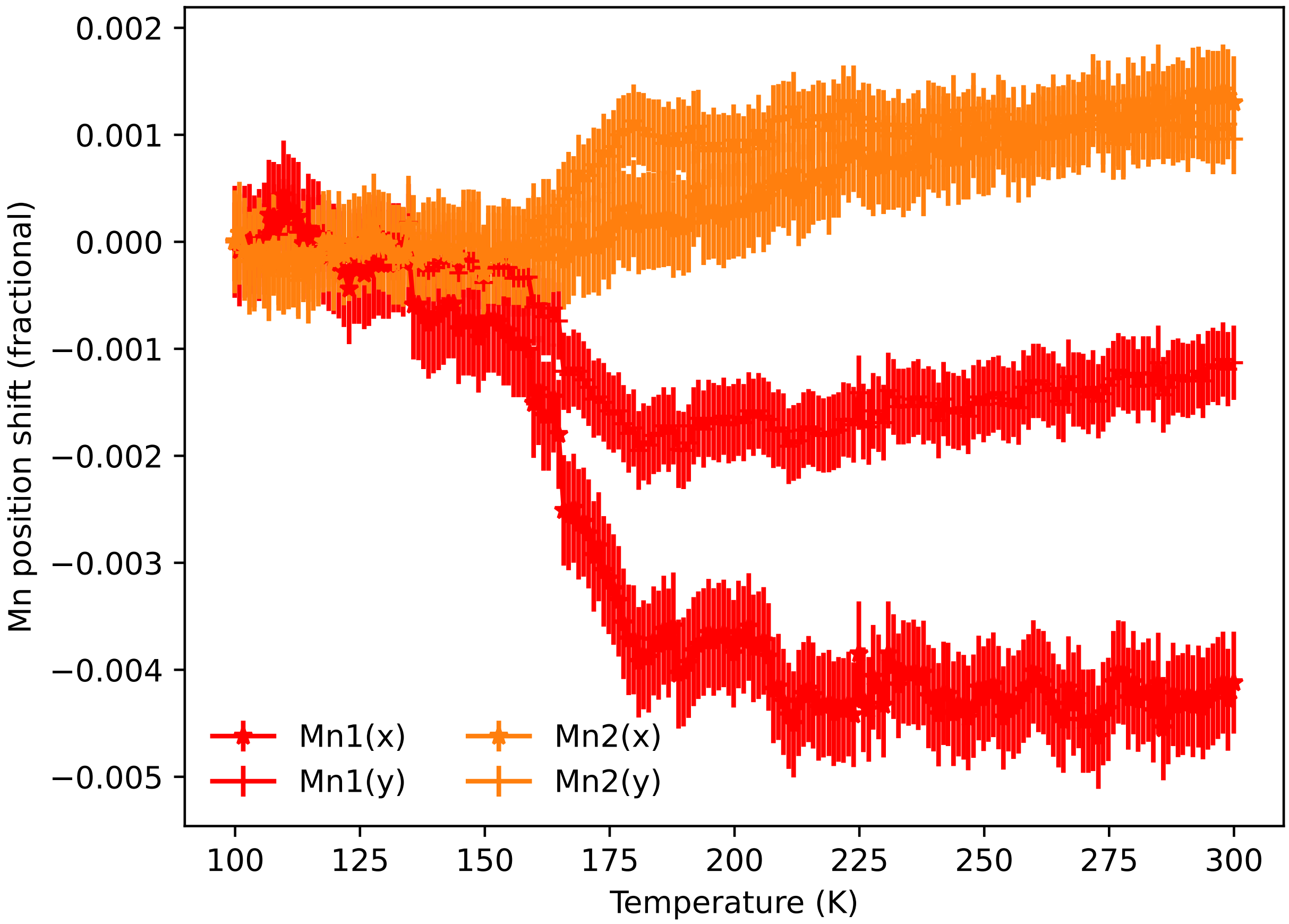

Figure S18: Shift in Mn1 and Mn2 atomic positions with temperature, as obtained by sequential Rietveld refinement of diffraction data (measured on KMO-S2) assuming a monoclinic unit cell, where diffraction patterns are analyzed in ascending order of temperature.

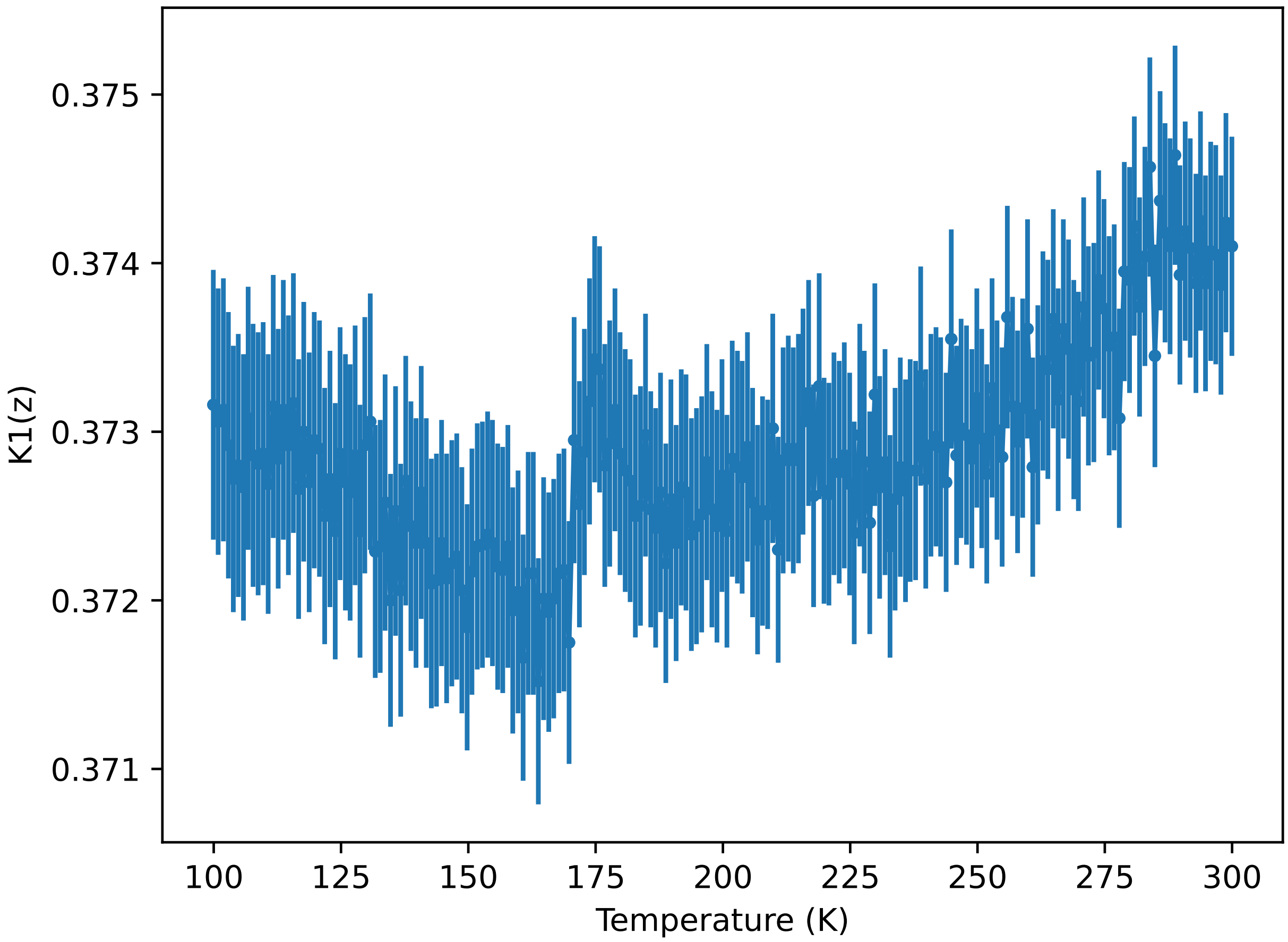

Figure S19: K atomic position with temperature, as obtained by sequential Rietveld refinement of diffraction data (measured on KMO-S2) assuming a monoclinic unit cell, where diffraction patterns are analyzed in ascending order of temperature.

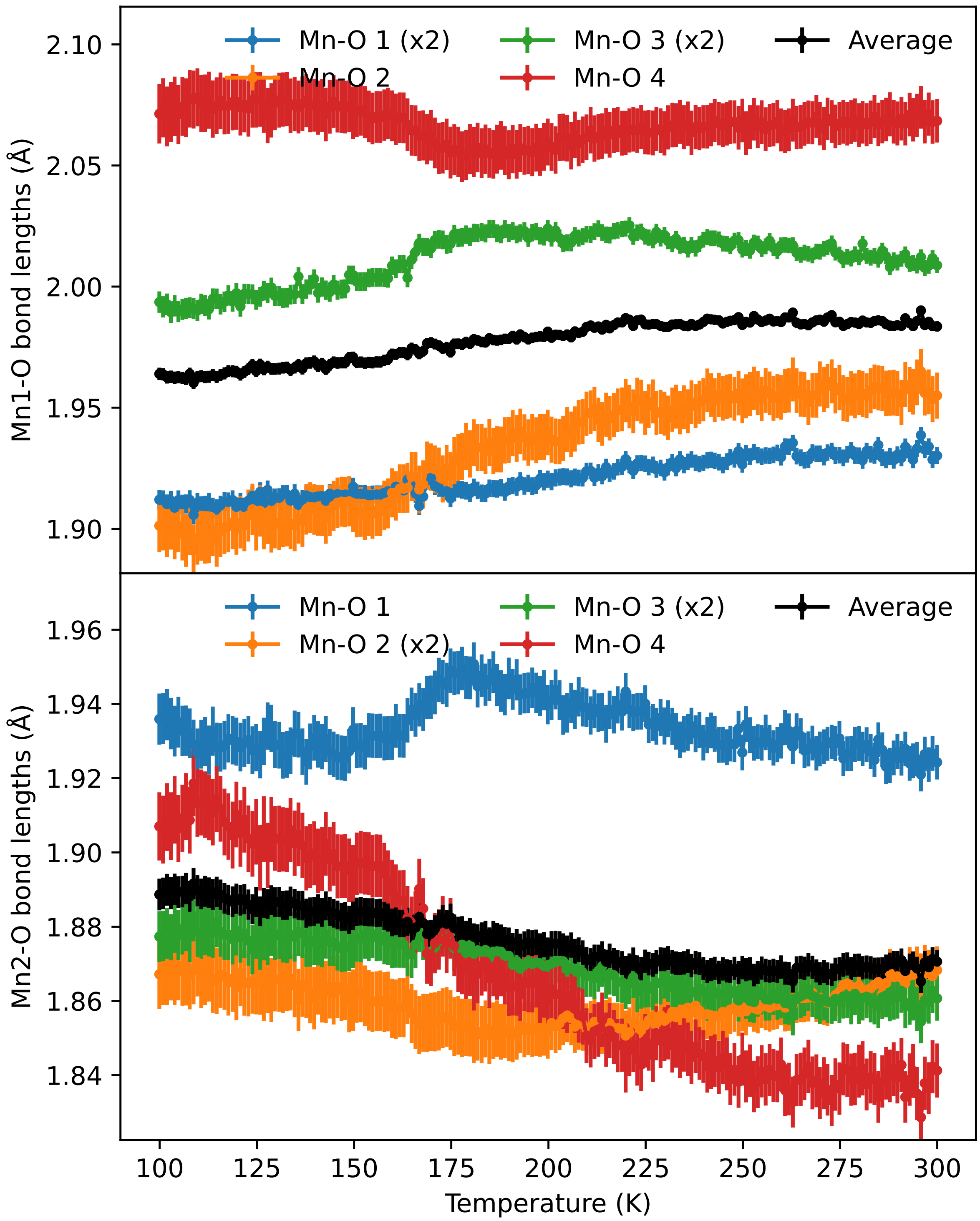

Figure S20: Dependence of Mn–O bond lengths on temperature for the two unique Mn sites in the unit cell, as obtained by sequential Rietveld refinement of diffraction data (measured on KMO-S2) assuming a monoclinic unit cell, where diffraction patterns are analyzed in ascending order of temperature.

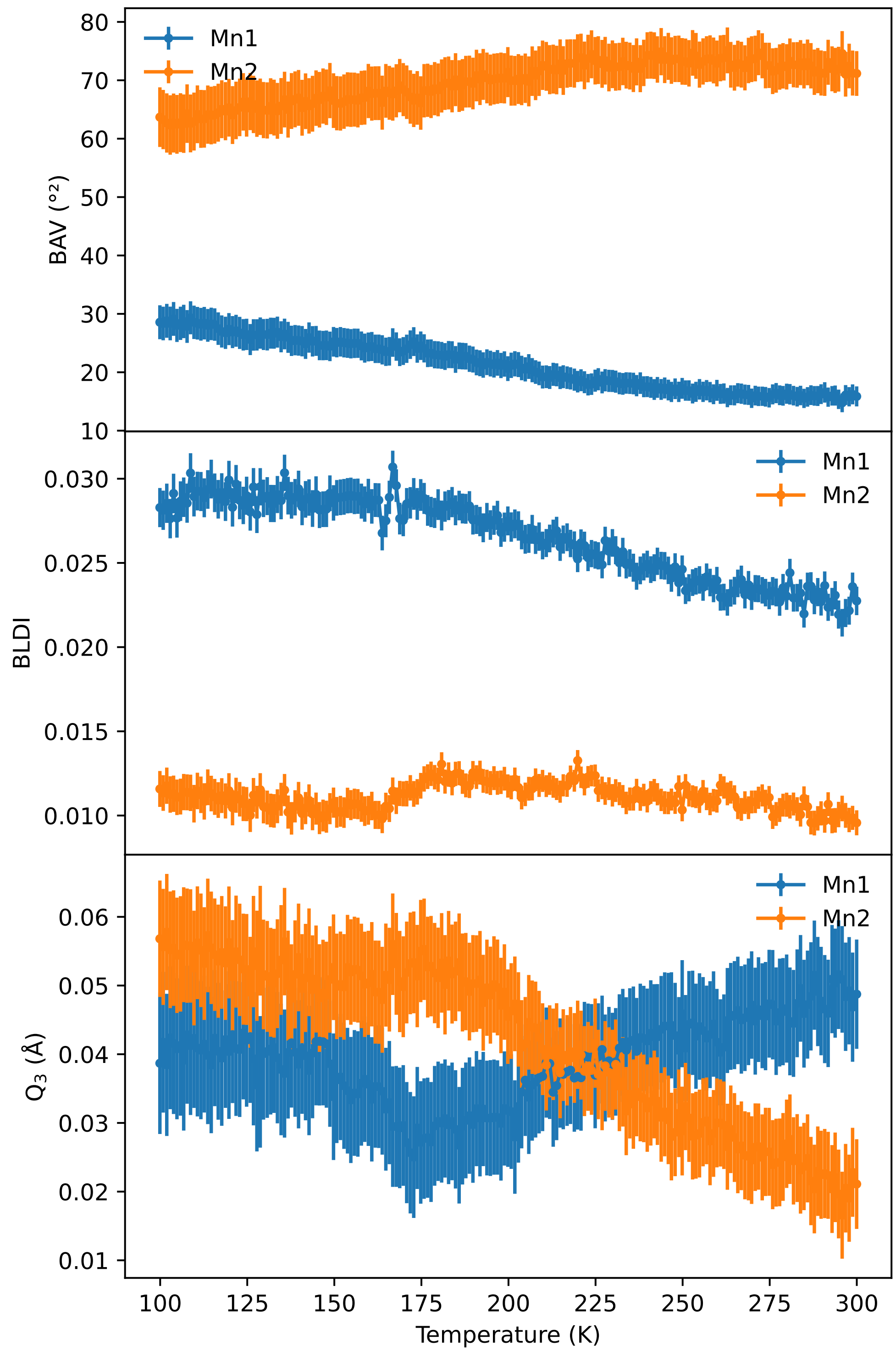

Figure S21: MnO_6 octahedral distortion (bond length distortion index, bond angle variance, and van Vleck Q_3 mode) as a function of temperature, as obtained by sequential Rietveld refinement of diffraction data (measured on KMO-S2) assuming a monoclinic unit cell, where diffraction patterns are analyzed in ascending order of temperature.

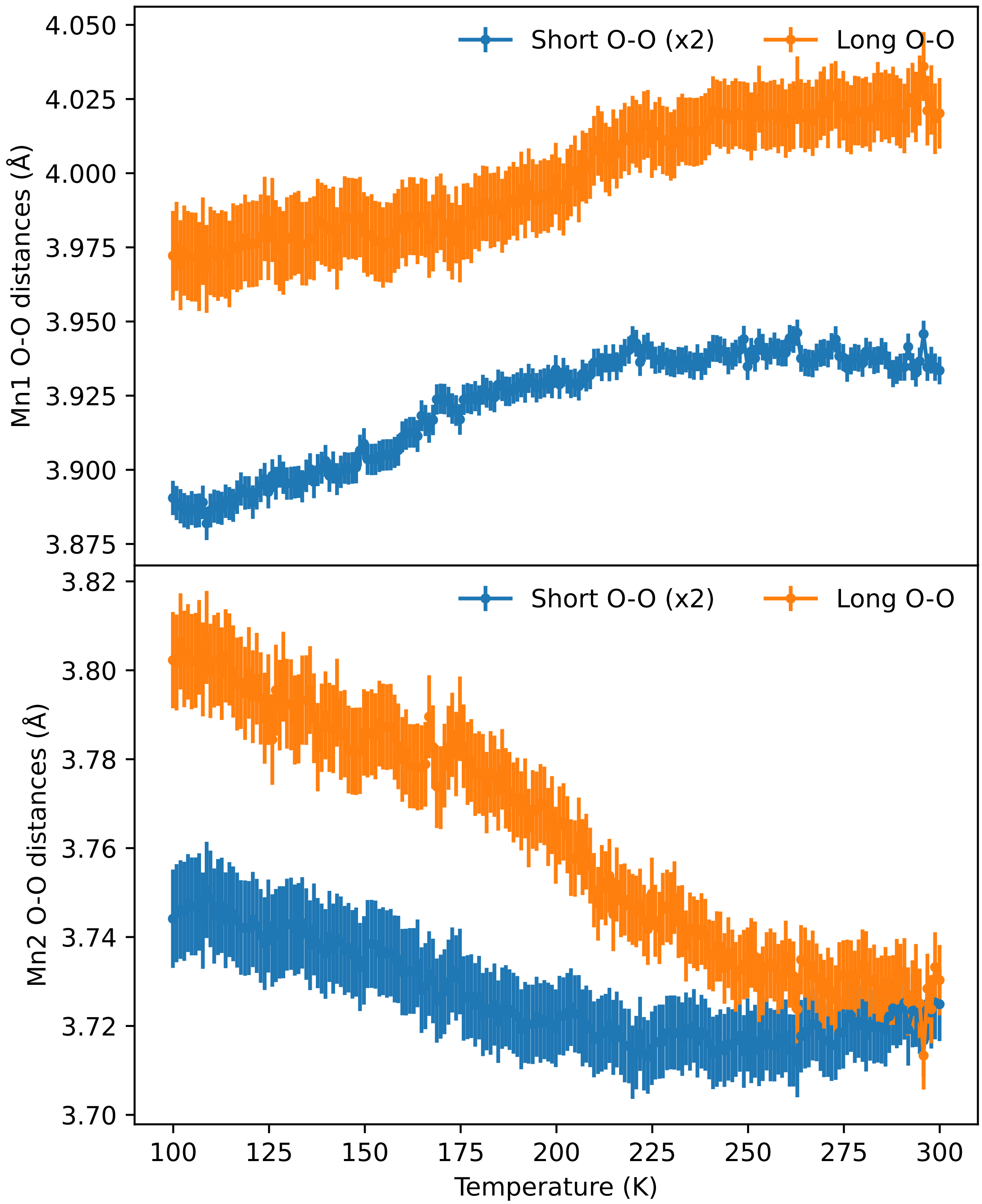

Figure S22: Equatorial O–O distances for both MnO_6 octahedra as a function of temperature, as obtained by sequential Rietveld refinement of diffraction data (measured on KMO-S2) assuming a monoclinic unit cell, where diffraction patterns are analyzed in ascending order of temperature.

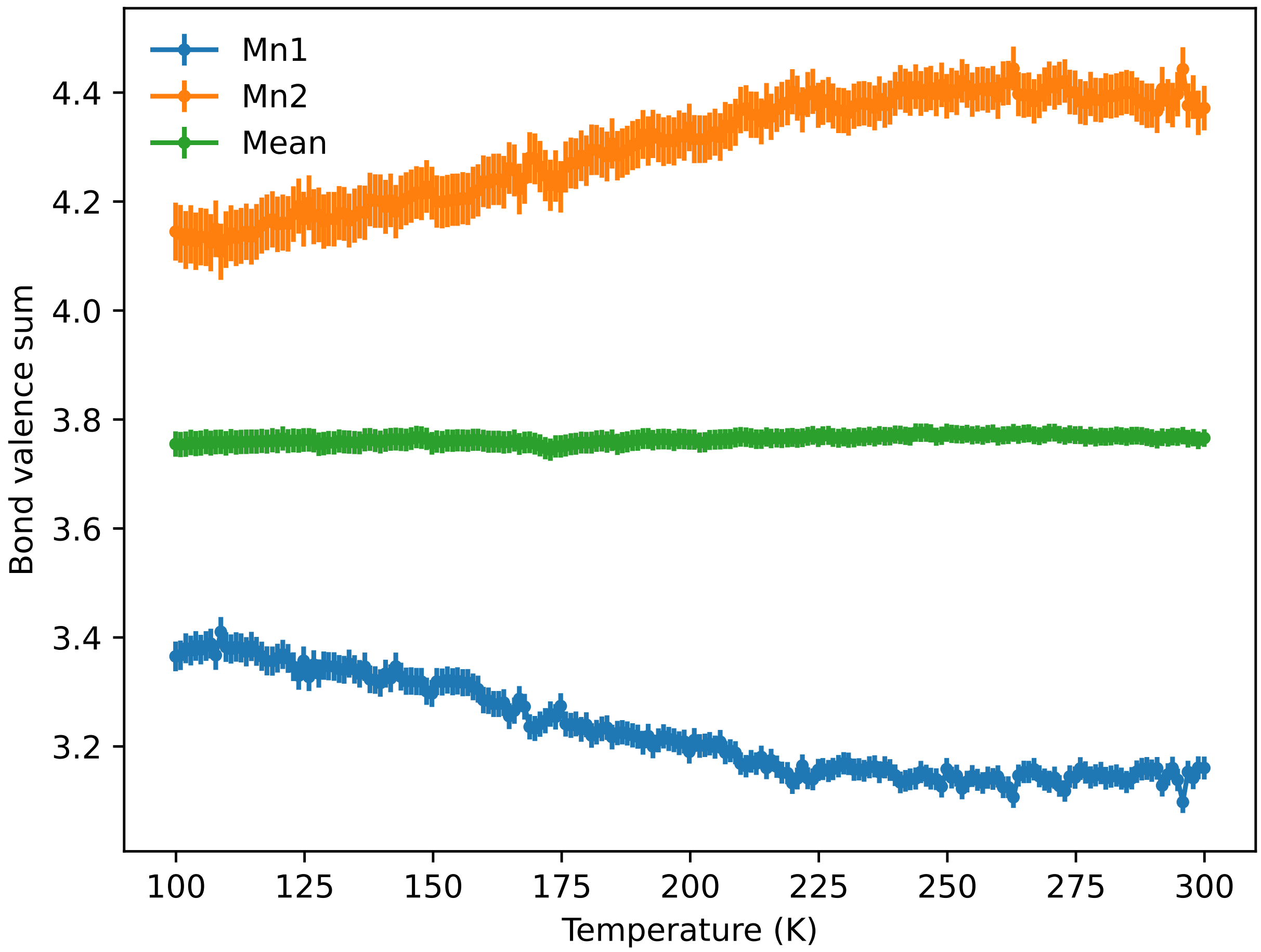

Figure S23: Bond valence sum of the two Mn sites, assuming $R_0 = 1.762$ Å and $B_0 = 0.34$, as obtained by sequential Rietveld refinement of diffraction data (measured on KMO-S2) assuming a monoclinic unit cell, where diffraction patterns are analyzed in ascending order of temperature.

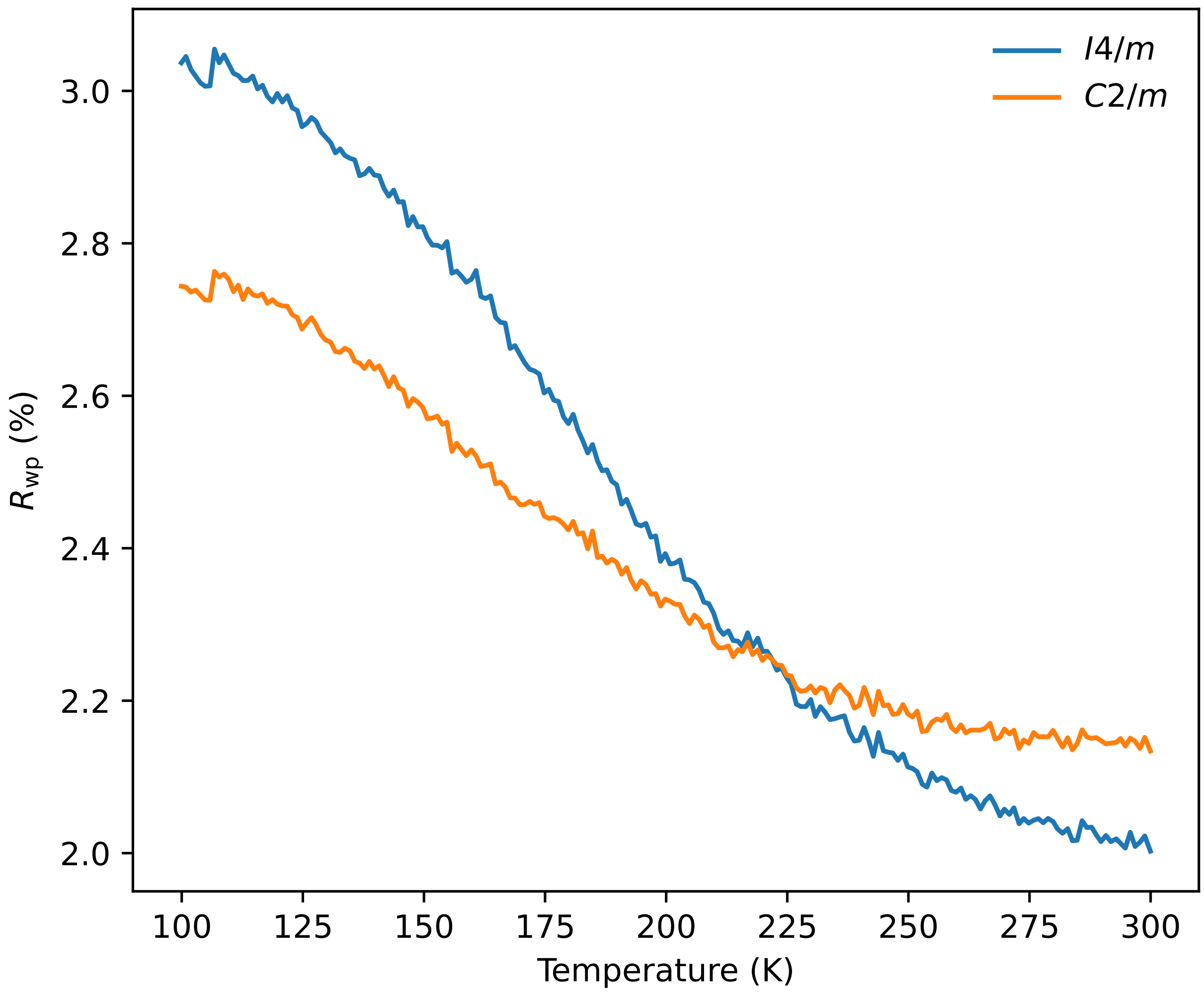

Figure S24: R_{wp} as a function of temperature in the sequential Rietveld refinement of diffraction data (measured on KMO-S2), comparing the result of the sequential refinement with a monoclinic (which was refined in $C2/m$ but we present using the alternate $A2/m$ setting elsewhere) and tetragonal ($I4/m$) unit cell. In both cases, diffraction patterns are analyzed in ascending order of temperature.

4.3.3. Sequential refinement of synchrotron data: tetragonal cell

As we believe that the tetragonal cell is sufficient to describe the crystal structure of cryptomelane above T_1, we have also analyzed this variable-temperature synchrotron diffraction data from I11 (Diamond Light Source) using the reported tetragonal $I4/m$ cell ($a = b$,$\alpha = \beta = \gamma = 90°$) which we use to fit the room-temperature diffraction data.

We have tested running the sequential Rietveld refinement with the tetragonal cell both starting with the low-temperature data, and starting with the high-temperature data (i.e. ascending or descending order), and find negligible difference between the two. All plots from the sequential refinement for the tetragonal cell are therefore presented for the case where diffraction patterns are analyzed in ascending order of temperature.

Figures S25 and S15 show the lattice parameters and cell volume as a function of temperature obtained by sequential Rietveld refinement. Figure S24 shows R_{wp} as a function of temperature for these Rietveld refinements compared to the refinement with the monoclinic cell. A better fit is obtained with the monoclinic cell at low temperatures, and the tetragonal cell at high temperatures, consistent with our interpretation that a phase transition occurs.

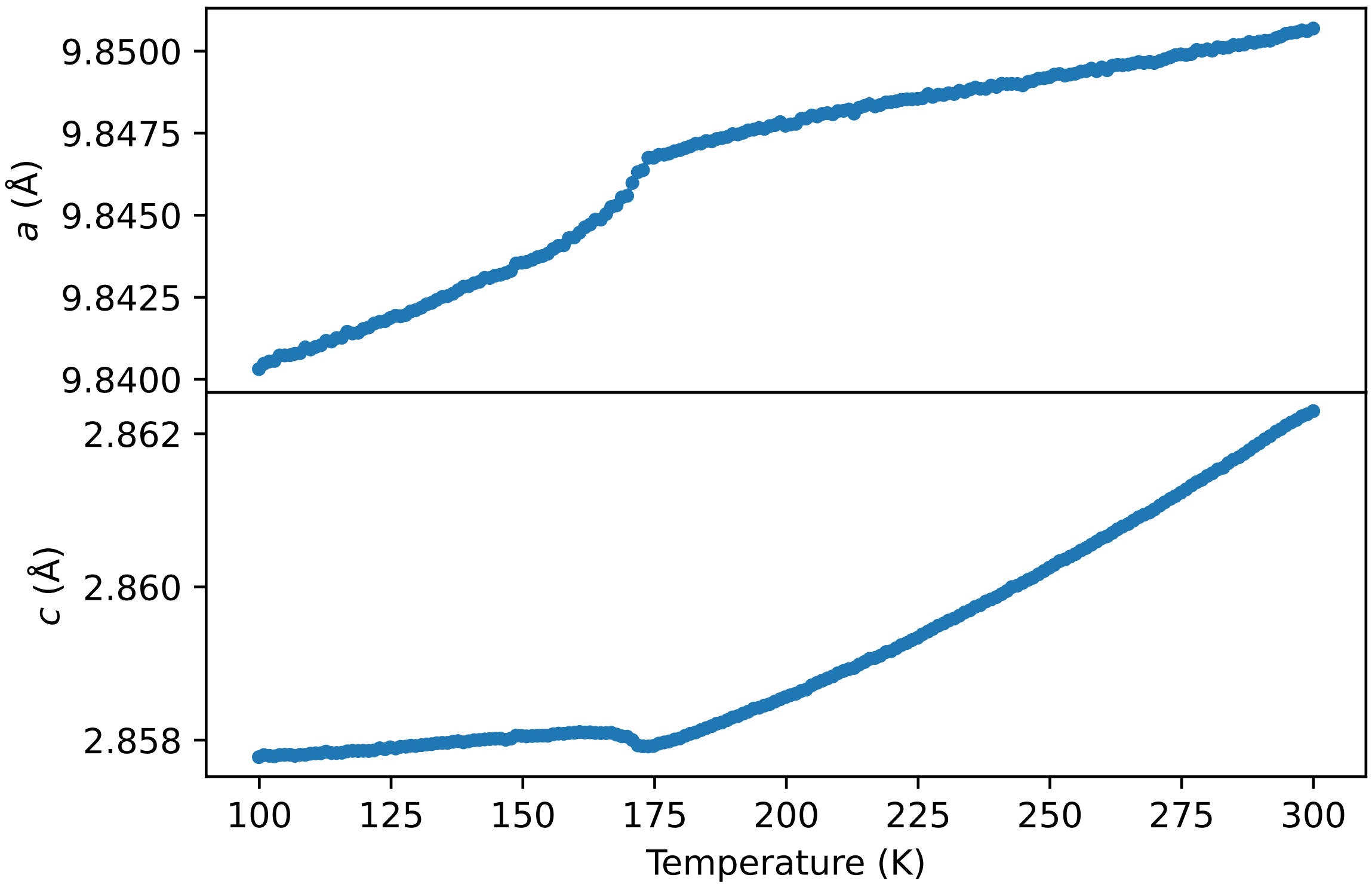

Figure S25: Refined unit cell parameters against temperature for tetragonal cryptomelane (KMO-S2) between 100 K and 300 K, as obtained by sequential Rietveld refinement of diffraction data. Here, diffraction patterns are analyzed in ascending order of temperature. Error bars are smaller than the data points.

4.3.4. *Indexing of the incommensurate structural propagation vector*

Here we determine k_z in $\vec{k}_{\text{struc}} = (0, 0, k_z)$, and its uncertainty, via a simultaneous fit of a series of pseudo-Voigt peaks to the incommensurate peaks, varying a linear background term, a peak intensity, and a peak width for each peak, and a single shared k_z. We perform this for a range of temperature points and use the resulting temperature series to test explicitly for temperature dependence and to report indexing residuals.

We label the same four reflections introduced above P1–P4, in order of decreasing d-spacing: $(0, \bar{1}, 1 - k_z)$, $(\bar{1}, 0, k_z)$, $(0, 0, k_z)$, and $(\bar{2}, 0, k_z)$, at $d \approx 7.35$, 3.37, 3.03, and 2.58 Å respectively. At each temperature, all of P1–P4 were fit simultaneously, each with its own pseudo-Voigt profile plus local linear background, using the raw measured intensity within a narrow window around the peak, with k_z shared across all four. Fits were performed using SciPy [5] and NumPy [3].

At 99.92 K, this joint fit gives $k_z = 0.74085(4)$.

To test explicitly for temperature dependence, we repeated this joint fit at each of the 51 temperature points of the sequential Rietveld refinement described in Section 4.3.2, spanning 99.92 K to 149.77 K, using the lattice parameters, zero-point, and wavelength refined at each temperature. The resulting $k_z(T)$ is shown in Figure S27. A weighted linear fit gives a slope of $(0.28 \pm 0.49) \times 10^{-6}\,\text{K}^{-1}$ (0.6σ from zero), i.e. consistent with zero, and we find no evidence for temperature dependence of k_z over this range. The weighted mean is $k_z = 0.740834(7)$, shown as the dashed line and gray uncertainty band in Figure S27.

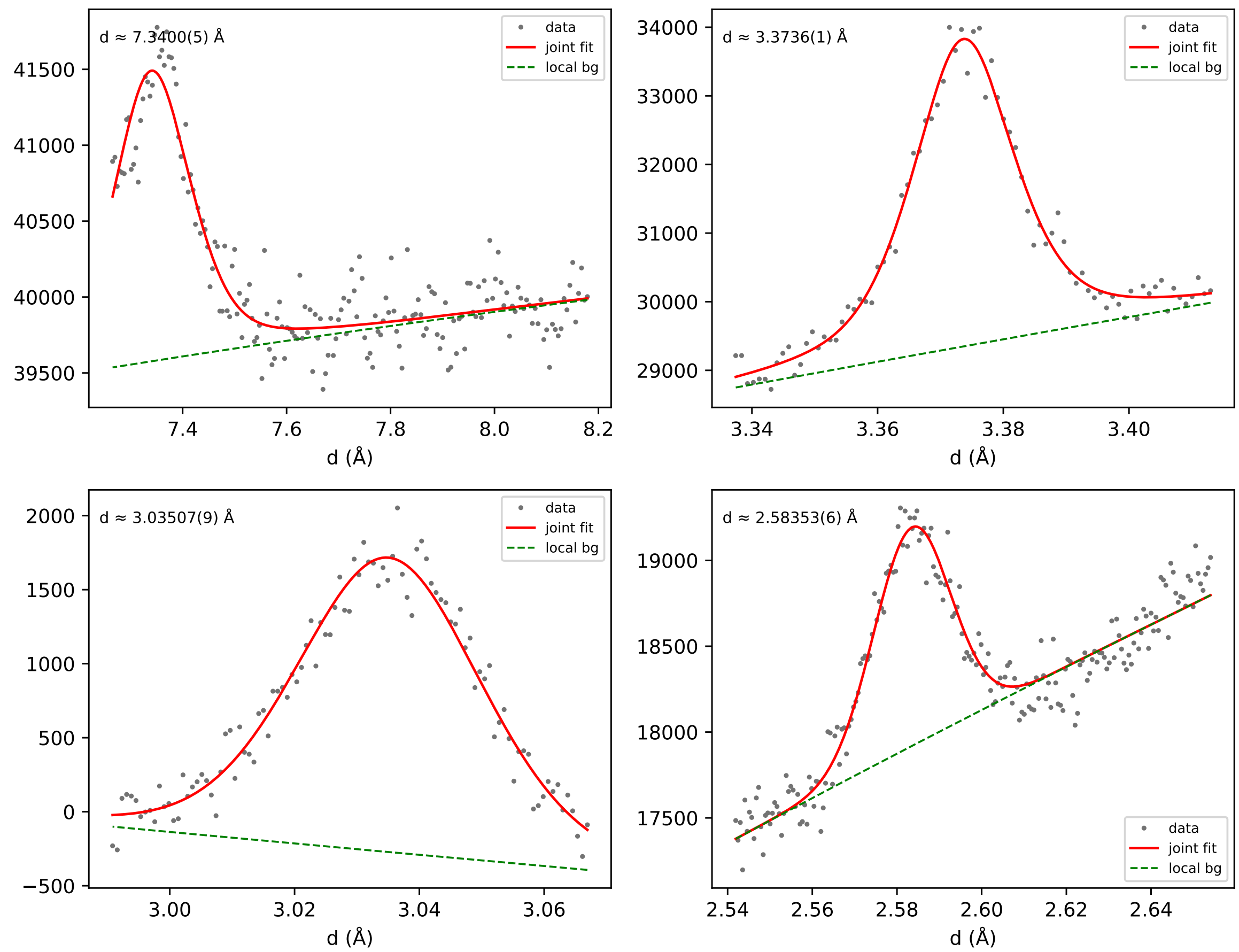

Figure S26:

Fits of the four incommensurate satellite peaks P1–P4 (from synchrotron diffraction data measured on KMO-S2 at $T = 99.92\,\mathrm{K}$) (top left to bottom right: $d \approx 7.34$, 3.37, 3.03, and 2.58 Å), used in the joint (Pawley-style) fit for k_z. Each panel shows the raw data, the pseudo-Voigt-plus-linear-background fit, and the local linear background; the x-axis has been converted from 2θ to d-spacing for display only (the fit itself was performed in 2θ). The annotated d-spacing and uncertainty in the top-left corner of each panel is the peak's fitted centroid, propagated through the shared, jointly-fitted uncertainty in k_z, rather than from the peak's own (uncorrelated) formal fit error, since k_z – not the four individual peak centroids – is the physically meaningful free parameter of the model.

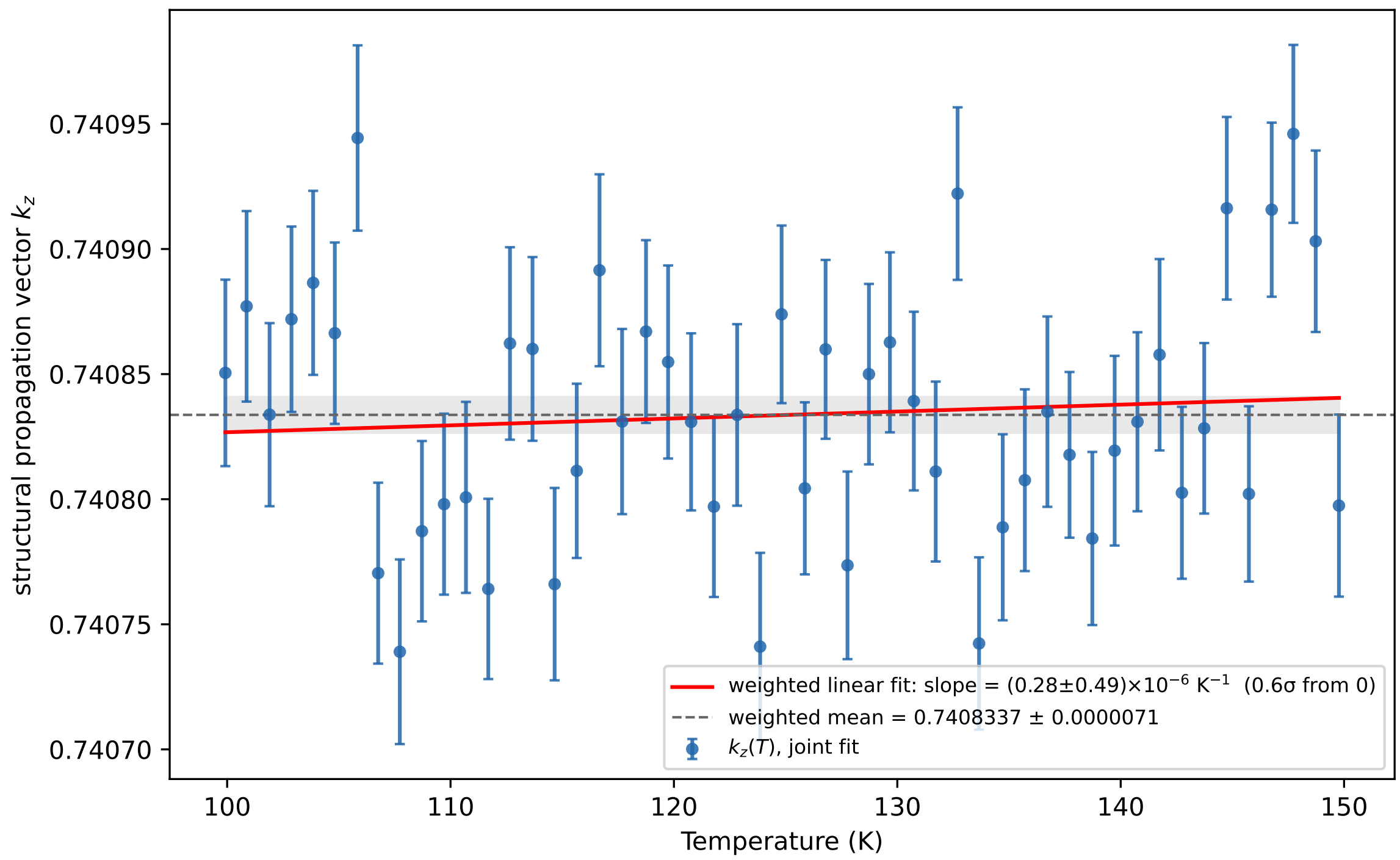

Figure S27: The structural propagation vector k_z as a function of temperature, from a joint (Pawley-style) fit of the four incommensurate satellite peaks P1–P4 (Figure S26) in the synchrotron diffraction data measured on KMO-S2 at each of 51 temperature points between 99.92 K and 149.77 K. Error bars are the reduced-χ^2-scaled joint-fit uncertainty in k_z at each temperature. The red line is a weighted linear fit, with slope $(0.28 \pm 0.49) \times 10^{-6}\,\mathrm{K}^{-1}$ (0.6σ from zero); the dashed line and gray band are the weighted mean, $k_z = 0.740834(7)$, and its uncertainty. We find no evidence for temperature dependence of k_z over this range, as the linear slope is zero within error.

5. OCTAHEDRAL DISTORTION PARAMETERS

5.1. Bond length distortion index

The bond length distortion index, D, for a polyhedron with a coordination number n (i.e. 6 for octahedra), is defined as:

$$D = \frac{1}{n}\sum_{i=1}^{n}\frac{|l_i - l_{\mathrm{av}}|}{l_{\mathrm{av}}} \tag{2}$$

where l_i is the distance between the core cation and the ith coordinated anion, and l_{av} is the average of all the distances between the core cation and coordinated anions [7].

5.2. Bond angle variance

Bond angle variance, σ^2, is defined as:

$$\sigma^2 = \frac{1}{m-1}\sum_{i=1}^{m}(\phi_i - \phi_0)^2 \tag{3}$$

where m is the number of bond angles (i.e. 12 for octahedra), ϕ_i is the ith bond angle, and ϕ_0 is the ideal bond angle for a regular polyhedron (i.e. 90 ° for an octahedron).

5.3. Q_3 mode

The Q_3 mode is one of the van Vleck modes [8, 9] of distortion of an octahedron, and is calculated as:

$$Q_3 = \frac{1}{\sqrt{3}} \times \frac{1}{2}\Big[(\bar{d} - d_+^x) + (\bar{d} - d_-^x) + (\bar{d} - d_+^y) + (\bar{d} - d_-^y) - 2\big((\bar{d} - d_+^z) + (\bar{d} - d_-^z)\big)\Big] \tag{4}$$

where $\bar{d}$ is the average of all 6 Mn-O bond lengths, and d_+^x, d_-^x, d_+^y, d_-^y, d_+^z, and d_-^z are the Mn-O bond lengths along the positive and negative Cartesian axes. This mathematical approach follows the Kanamori approximation [10] by which angular distortion is disregarded and bonds assumed to be perpendicular.

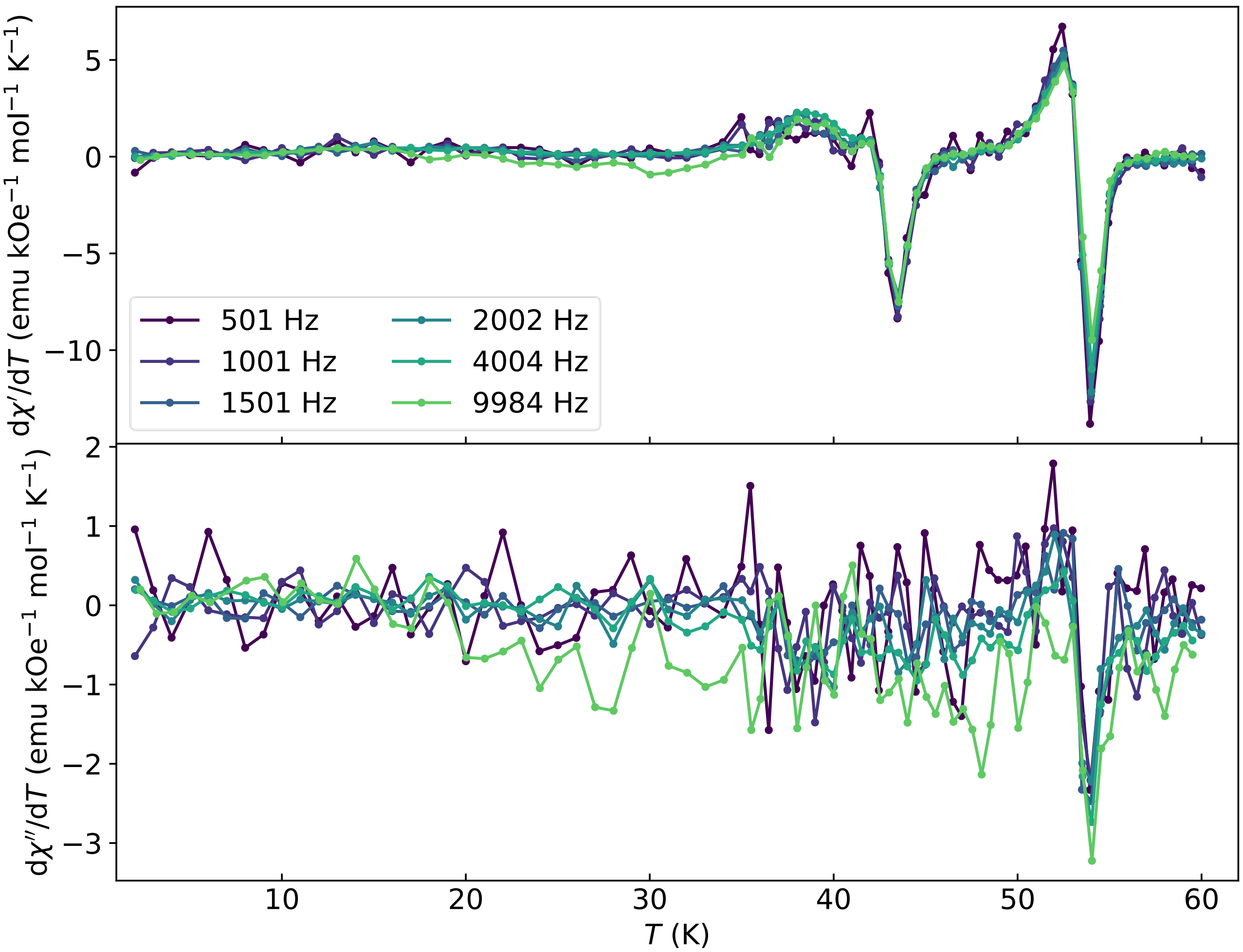

Figure S28: Derivative of AC susceptibility with temperature (KMO-S1) for various frequencies: 501 Hz, 1001 Hz, 1501 Hz, 2002 Hz, 4004 Hz, and 9984 Hz, shown in terms of the real χ' (top) and imaginary χ'' (bottom) components of the overall AC susceptibility.

6. AC SUSCEPTIBILITY: DERIVATIVE WITH TEMPERATURE

In the main paper, we present frequency- and temperature-dependent AC susceptibility data, in terms of the real χ' and imaginary χ'' component. The derivative of susceptibility with temperature, $\mathrm{d}\chi/\mathrm{d}T$, is generally informative for assigning transition temperatures and identifying subtle transitions. However, in this case we have excellent DC susceptibility data (presented in the manuscript), and the AC $\mathrm{d}\chi/\mathrm{d}T$ does not show any features which yields useful information. For this reason, we exclude it from the manuscript, but include it here in Supplementary Information. It was calculated using the NumPy [3] function GRADIENT, and is presented in Figure S28, for the following frequencies: 501 Hz, 1001 Hz, 1501 Hz, 2002 Hz,

4004 Hz, and 9984 Hz.

7. MAGNETIC MODELS USED FOR NEUTRON DIFFRACTION ANALYSIS

In this section of the SI, we detail the mathematics behind the representational analysis used to test different models of the commensurate and incommensurate magnetism of cryptomelane.

For a given site symmetry and propagation vector $\vec{k}_{\text{mag}}$, there are several possible Irreducible Representations (IR) which describe a possible configuration of the basis vectors on each site containing a magnetic atom. These are determined with BASIREPS [11].

7.1. Monoclinic IRs

The main magnetic analysis performed here assumes the monoclinic cell determined below T_1 based on the synchrotron diffraction data. This cell symmetry contains two unique Mn sites, both described by Wyckoff notation $4i(x,\ 0,\ z)$. This section retains the conventional $C2/m$ setting (unique axis b) throughout, including the propagation vector components and Wyckoff site description below, as this is the setting in which the BASIREPS representational analysis was performed; elsewhere in this work the equivalent $A2/m$ setting (unique axis c) is used for consistency with the $I4/m$ parent cell. All fits are presented in the $A2/m$ setting.

The IRs determined for the relevant propagation vectors and the $4i$ Wyckoff site of the monoclinic ($C2/m$) cryptomelane unit cell are described here. There are 2 IRs for $\vec{k}_{\text{mag}} = (0, \sim 0.37, 0)$ and 4 IRs for $\vec{k}_{\text{mag}} = (0, 0, 0)$; these are denoted using Γ_i^j ($i = 1, 2, 3,$ or 4; $j =$ IC or C where IC/C stands for InCommensurate or Commensurate).

The components C_i for the basis vectors ψ_i are tabulated below. Table S5 shows the basis vectors for the IRs of $\vec{k}_{\text{mag}} = (0, \sim 0.37, 0)$ and Table S6 shows the basis vectors for the IRs of $\vec{k}_{\text{mag}} = (0, 0, 0)$.

IR	BV	Atom	BV components					
			$m_{\|a}$	$m_{\|b}$	$m_{\|c}$	$im_{\|a}$	$im_{\|b}$	$im_{\|c}$
Γ_1	ψ_1	(x, y, z)	1	0	0	0	0	0
		$(-x, y, -z)$	-1	0	0	0	0	0
	ψ_2	(x, y, z)	0	1	0	0	0	0
		$(-x, y, -z)$	0	1	0	0	0	0
	ψ_3	(x, y, z)	0	0	1	0	0	0
		$(-x, y, -z)$	0	0	-1	0	0	0
Γ_2	ψ_1	(x, y, z)	1	0	0	0	0	0
		$(-x, y, -z)$	1	0	0	0	0	0
	ψ_2	(x, y, z)	0	1	0	0	0	0
		$(-x, y, -z)$	0	-1	0	0	0	0
	ψ_3	(x, y, z)	0	0	1	0	0	0
		$(-x, y, -z)$	0	0	1	0	0	0

TABLE S5: Basis vectors for the monoclinic ($C2/m$) cell with $\vec{k}_{\text{mag}} = (0, 0.37, 0)$. The table uses the notation of Kovalev [12]. Imaginary components are all zero. As discussed elsewhere, the irreps were generated using a conventional special b-axis, with the fits and reported parameters later presented with special c-axis for consistency with the room-temperature tetragonal structure.

IR	BV	Atom	BV components					
			$m_{\parallel a}$	$m_{\parallel b}$	$m_{\parallel c}$	$im_{\parallel a}$	$im_{\parallel b}$	$im_{\parallel c}$
Γ_1	ψ_1	(x, y, z)	0	1	0	0	0	0
		$(-x, y, -z)$	0	1	0	0	0	0
Γ_2	ψ_1	(x, y, z)	1	0	0	0	0	0
		$(-x, y, -z)$	-1	0	0	0	0	0
	ψ_2	(x, y, z)	0	0	1	0	0	0
		$(-x, y, -z)$	0	0	-1	0	0	0
Γ_3	ψ_1	(x, y, z)	1	0	0	0	0	0
		$(-x, y, -z)$	1	0	0	0	0	0
	ψ_2	(x, y, z)	0	0	1	0	0	0
		$(-x, y, -z)$	0	0	1	0	0	0
Γ_4	ψ_1	(x, y, z)	0	1	0	0	0	0
		$(-x, y, -z)$	0	-1	0	0	0	0

TABLE S6: Basis vectors for the monoclinic ($C2/m$) cell with $\vec{k}_{\mathrm{mag}} = (0, 0, 0)$. The table uses the notation of Kovalev [12]. Imaginary components are all zero. As discussed elsewhere, the irreps were generated using a conventional special *b*-axis, with the fits and reported parameters later presented with special *c*-axis for consistency with the room-temperature tetragonal structure.

7.2. Tetragonal IRs

Although we believe based on the synchrotron data that the unit cell at low temperatures is actually monoclinic ($A2/m$), we cannot distinguish between a monoclinic and tetragonal ($I4/m$) cell from the neutron diffraction data from the D20 instrument. We therefore also perform the magnetic neutron diffraction analysis using the tetragonal cell, which has a single Mn $8h(x,\ y,\ 0)$ site.

The IRs determined for the relevant propagation vectors and the tetragonal ($I4/m$) cryptomelane unit cell are described here. There are 4 IRs for $\vec{k}_{\text{mag}} = (0, 0, \sim 0.37)$ and 6 IRs for $\vec{k}_{\text{mag}} = (0, 0, 0)$ on the $8h$ Wyckoff site of the $I4/m$ cell; these are denoted using Γ_i^j ($i = 1, 2, 3, 4, 5,$ or 6; $j =$ IC or C where IC/C stands for InCommensurate or Commensurate).

The components C_i for the basis vectors ψ_i are tabulated below. Table S7 shows the basis vectors for the IRs of $\vec{k}_{\text{mag}} = (0, 0, \sim 0.37)$ and Table S8 shows the basis vectors for the IRs of $\vec{k}_{\text{mag}} = (0, 0, 0)$.

7.3. Phase shifts

Additionally, each basis vector (BV) within an IR can have a phase shift, ϕ, (in this study, either 0 or $\frac{\pi}{2}$ radians) applied to it, which defines the relative phase between the basis vectors. The presence of a relative phase between basis vectors is used to simulate helical or cycloidal magnetic structures depending on whether the rotation occurs around, or in the plane of, the propagation vector respectively. The absence of phase shift would indicate entirely sinusoidal modulations.

Phase shifts are achieved within FullProf [13] by setting the real/imaginary indicators for the basis vector coefficients as zero (no phase shift) or 1 ($\frac{\pi}{2}$ radian phase shift).

IR	BV	Atom	BV components						IR	BV	Atom	BV components					
			$m_{\parallel a}$	$m_{\parallel b}$	$m_{\parallel c}$	$im_{\parallel a}$	$im_{\parallel b}$	$im_{\parallel c}$				$m_{\parallel a}$	$m_{\parallel b}$	$m_{\parallel c}$	$im_{\parallel a}$	$im_{\parallel b}$	$im_{\parallel c}$
Γ_1	ψ_1	x,y,z	1	0	0	0	0	0	Γ_3	ψ_1	x,y,z	1	0	0	0	0	0
		$-x,-y,z$	-1	0	0	0	0	0			$-x,-y,z$	1	0	0	0	0	0
		$-y,x,z$	0	1	0	0	0	0			$-y,x,z$	0	0	0	0	-1	0
		$y,-x,z$	0	-1	0	0	0	0			$y,-x,z$	0	0	0	0	-1	0
	ψ_2	x,y,z	0	1	0	0	0	0		ψ_2	x,y,z	0	1	0	0	0	0
		$-x,-y,z$	0	-1	0	0	0	0			$-x,-y,z$	0	1	0	0	0	0
		$-y,x,z$	-1	0	0	0	0	0			$-y,x,z$	0	0	0	1	0	0
		$y,-x,z$	1	0	0	0	0	0			$y,-x,z$	0	0	0	1	0	0
	ψ_3	x,y,z	0	0	1	0	0	0		ψ_3	x,y,z	0	0	1	0	0	0
		$-x,-y,z$	0	0	1	0	0	0			$-x,-y,z$	0	0	-1	0	0	0
		$-y,x,z$	0	0	1	0	0	0			$-y,x,z$	0	0	0	0	0	-1
		$y,-x,z$	0	0	1	0	0	0			$y,-x,z$	0	0	0	0	0	1
Γ_2	ψ_1	x,y,z	1	0	0	0	0	0	Γ_4	ψ_1	x,y,z	1	0	0	0	0	0
		$-x,-y,z$	-1	0	0	0	0	0			$-x,-y,z$	1	0	0	0	0	0
		$-y,x,z$	0	-1	0	0	0	0			$-y,x,z$	0	0	0	0	1	0
		$y,-x,z$	0	1	0	0	0	0			$y,-x,z$	0	0	0	0	1	0
	ψ_2	x,y,z	0	1	0	0	0	0		ψ_2	x,y,z	0	1	0	0	0	0
		$-x,-y,z$	0	-1	0	0	0	0			$-x,-y,z$	0	1	0	0	0	0
		$-y,x,z$	1	0	0	0	0	0			$-y,x,z$	0	0	0	-1	0	0
		$y,-x,z$	-1	0	0	0	0	0			$y,-x,z$	0	0	0	-1	0	0
	ψ_3	x,y,z	0	0	1	0	0	0		ψ_3	x,y,z	0	0	1	0	0	0
		$-x,-y,z$	0	0	1	0	0	0			$-x,-y,z$	0	0	-1	0	0	0
		$-y,x,z$	0	0	-1	0	0	0			$-y,x,z$	0	0	0	0	0	1
		$y,-x,z$	0	0	-1	0	0	0			$y,-x,z$	0	0	0	0	0	-1

TABLE S7: Basis vectors for the tetragonal ($I4/m$) cell with $\vec{k}_{\rm mag} = (0, 0,\ 0.37)$. The table uses the notation of Kovalev [12].

IR	BV	Atom	BV components			IR	BV	Atom	BV components		
			$m_{\parallel a}$	$m_{\parallel b}$	$m_{\parallel c}$				$m_{\parallel a}$	$m_{\parallel b}$	$m_{\parallel c}$
Γ_1	ψ_1	x,y,z	0	0	1	Γ_4	ψ_1	x,y,z	1	0	0
		$-x$,$-y$,z	0	0	1			$-x$,$-y$,z	-1	0	0
		$-y$,x,z	0	0	1			$-y$,x,z	0	1	0
		y,$-x$,z	0	0	1			y,$-x$,z	0	-1	0
Γ_2	ψ_1	x,y,z	0	0	1		ψ_2	x,y,z	0	1	0
		$-x$,$-y$,z	0	0	1			$-x$,$-y$,z	0	-1	0
		$-y$,x,z	0	0	-1			$-y$,x,z	-1	0	0
		y,$-x$,z	0	0	-1			y,$-x$,z	1	0	0
Γ_3	ψ_1	x,y,z	1	0	0	Γ_5	ψ_1	x,y,z	1	0	0
		$-x$,$-y$,z	1	0	0			$-x$,$-y$,z	-1	0	0
		$-y$,x,z	0	0	0			$-y$,x,z	0	-1	0
		y,$-x$,z	0	0	0			y,$-x$,z	0	1	0
	ψ_2	x,y,z	0	1	0		ψ_2	x,y,z	0	1	0
		$-x$,$-y$,z	0	1	0			$-x$,$-y$,z	0	-1	0
		$-y$,x,z	0	0	0			$-y$,x,z	1	0	0
		y,$-x$,z	0	0	0			y,$-x$,z	-1	0	0
	ψ_3	x,y,z	0	0	0	Γ_6	ψ_1	x,y,z	0	0	1
		$-x$,$-y$,z	0	0	0			$-x$,$-y$,z	0	0	-1
		$-y$,x,z	0	1	0			$-y$,x,z	0	0	0
		y,$-x$,z	0	1	0			y,$-x$,z	0	0	0
	ψ_4	x,y,z	0	0	0		ψ_2	x,y,z	0	0	0
		$-x$,$-y$,z	0	0	0			$-x$,$-y$,z	0	0	0
		$-y$,x,z	-1	0	0			$-y$,x,z	0	0	1
		y,$-x$,z	-1	0	0			y,$-x$,z	0	0	-1

TABLE S8: Basis vectors for the tetragonal ($I4/m$) cell with $\vec{k}_{\text{mag}} = (0,0,0)$. Γ_3 was the IR used for $T_3 < T < T_2$. The table uses the notation of Kovalev [12]. Imaginary components are all zero so are not displayed.

8. MAGNETIC NEUTRON DIFFRACTION ANALYSIS FOR $T_3 < T < T_2$ ASSUMING MONOCLINIC CELL

In the main text of the paper, we present the analysis of the magnetic neutron diffraction data using the monoclinic cell. Specifically, we use a helical magnetic model, as shown in Figure 8 of the main paper.

8.1. Varying incommensurate models at 47 K

As noted, we use a helical model for most fitting, as this model is simple and does not result in zero-moment Mn which we find physically unreasonable. However, we also achieved a good fit using a sinusoidal magnetic structure (i.e. with a phase shift of 0 between the real and imaginary components of the basis vectors). The refined magnetic structure, and fit, for the sinusoidal case is shown in Figures S30 and S29 respectively. We found no dependence of fit quality on the plane in which the sinusoid occurs, and so the plane of oscillation in Figure S30 is arbitrary. For the sinusoidal model, we obtained a moment of 0.320(11) μ_{B} for the commensurate component, consistent with what we get for the helical fit presented in the main text.

Additionally, as a true helical (constant-moment) model is no longer symmetry-protected in the monoclinic cell, as compared with the tetragonal cell, we tested a variable-moment ellipsoidal helix, in which moments are equal in magnitude along the lattice directions of the cell, but these are 135° apart, leading to an ellipsoid. The precise geometry of this ellipsoid is arbitrary for the purposes of testing an ellipsoidal helix. We note that this model also yields an acceptable fit to the data, as shown in Figure S31. Despite presenting the helix in the main paper, we therefore cannot definitely state that the incommensurate component of the structure is indeed constant-moment based on the available experimental evidence. In any case, as the true magnetic structure is a superposition of the commensurate and incommensurate components, even if the incommensurate component is constant-moment helical, the true magnetic structure cants the helix in a particular direction.

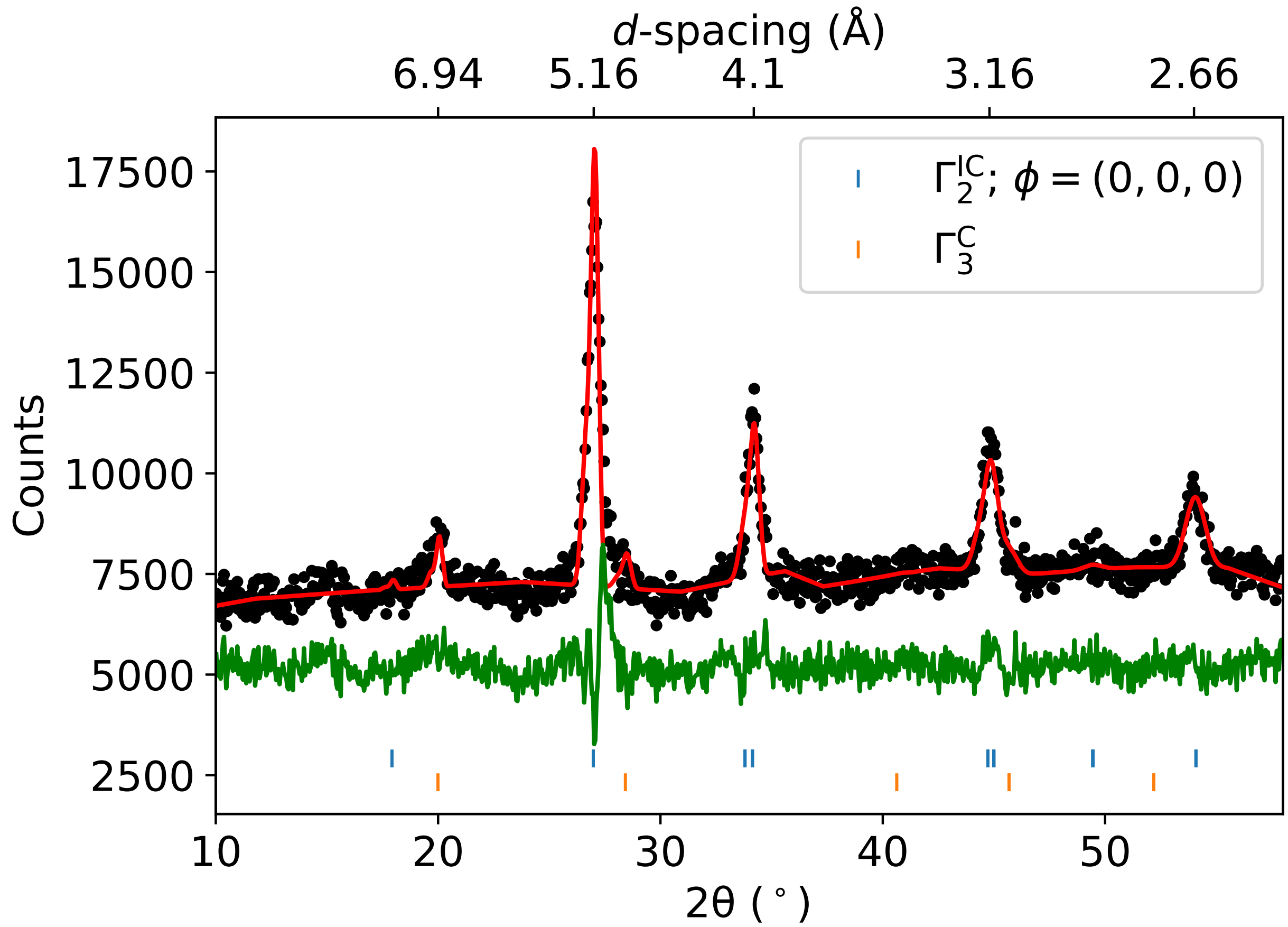

Figure S29: Experimental difference pattern 47 K-60 K (measured on KMO-S1 using D20 at ILL), compared with calculated pattern. The calculated pattern was obtained by Rietveld refinement of the model for $T_3 < T < T_2$, consisting of a commensurate $\vec{k}_{\rm mag} = 0$ and incommensurate $\vec{k}_{\rm mag} = (0, 0, \sim 0.37)$ component,assuming a monoclinic ($A2/m$) unit cell. The incommensurate structure was represented with a sinusoidal model.

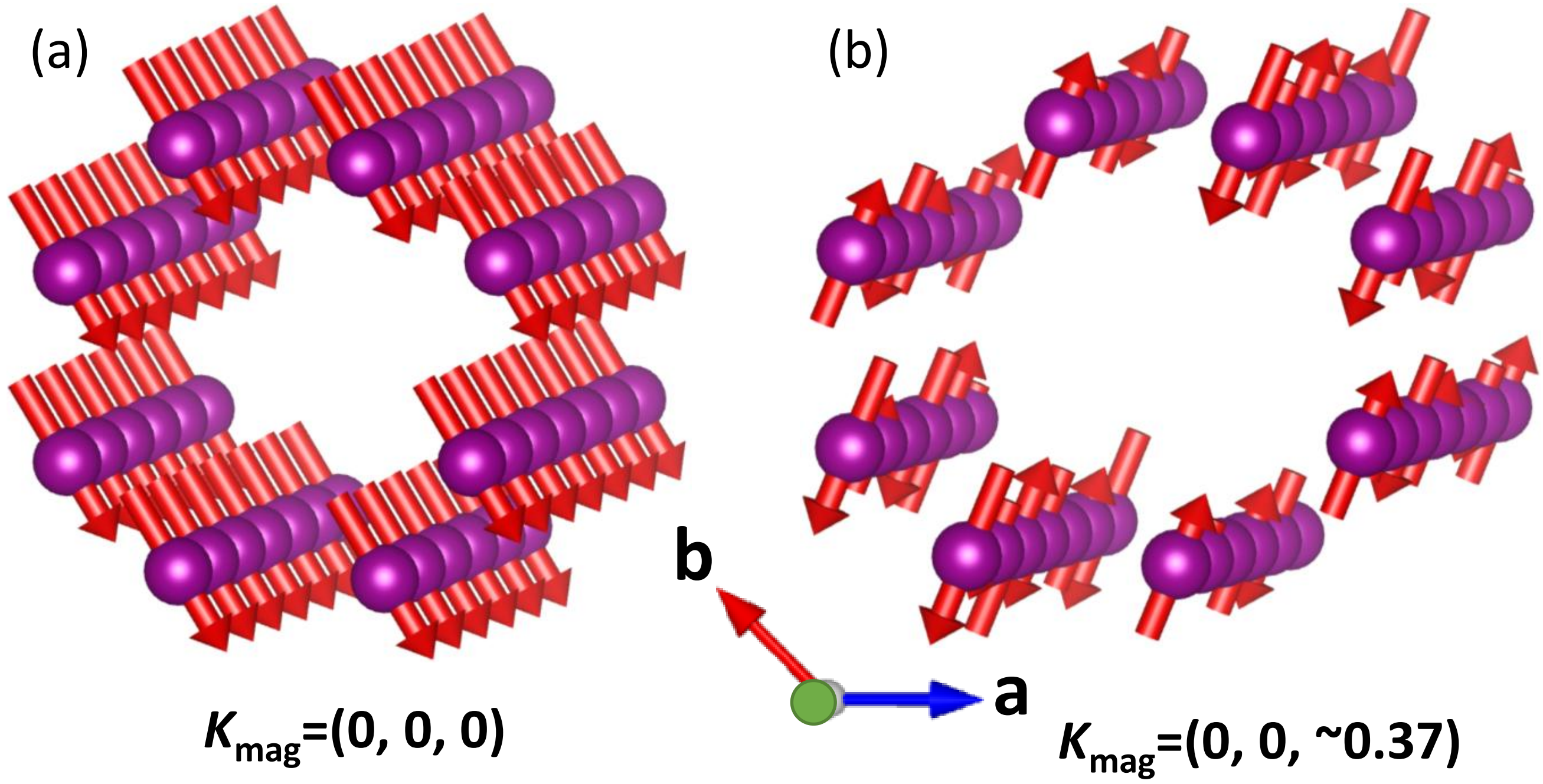

Figure S30: The refined magnetic unit cell for the (a) commensurate $\vec{k}_{\text{mag}} = 0$ and (b) incommensurate $\vec{k}_{\text{mag}} = (0, 0, 0.37)$ components of the magnetic structure for $T_3 < T < T_2$ assuming a monoclinic ($A2/m$) unit cell. Here we assume the modulated magnetism is sinusoidal, with the plane of oscillation arbitrary. We also assume the alignment of the commensurate moments arbitrarily.

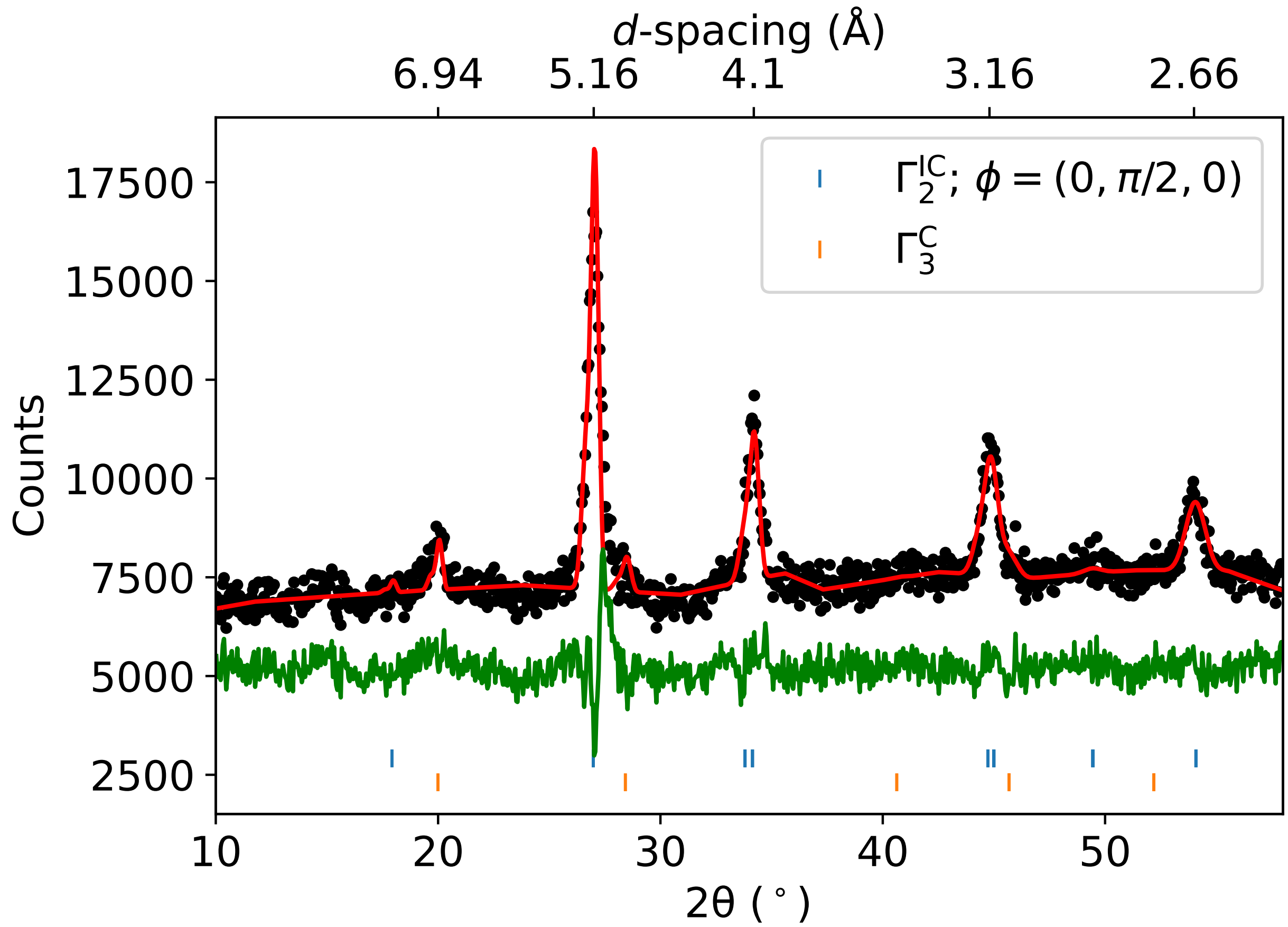

Figure S31: Experimental difference pattern 47 K-60 K (measured on KMO-S1 using D20 at ILL), compared with calculated pattern. The calculated pattern was obtained by Rietveld refinement of the model for $T_3 < T < T_2$, consisting of a commensurate $\vec{k}_{\text{mag}} = 0$ and incommensurate $\vec{k}_{\text{mag}} = (0, 0, \sim 0.37)$ component, a monoclinic ($A2/m$) unit cell. The incommensurate structure was represented with an ellipsoidal model, in which moments are equal in magnitude along the lattice directions of the cell, but these are 135 ° apart, leading to an ellipsoid.

8.2. Comparison to fits at 40 K and 44 K

We also performed magnetic Rietveld refinements against the difference patterns at $T =$ 44 K and $T = 40$ K, using the same monoclinic ($A2/m$) model as Figure 9 of the main text: a commensurate Γ_3^{C} component, and an incommensurate Γ_2^{IC} component represented as a helical model in which constant moments rotate about the propagation vector, achieved by representing moments as spherical coordinates and configuring a 180° phase shift between the two Mn sites. These fits are shown in Figures S32 and S33, with refined moment magnitudes given in Table S9. For the 40 K refinement, we additionally included a magnetic phase for the Mn_3O_4 impurity, with propagation vector $\vec{k}_{\mathrm{mag}} = (0, 0, 0)$ and an entirely collinear moment arrangement.

TABLE S9: Refined moment magnitudes (in μ_{B}) for the commensurate (Γ_3^{C}) and incommensurate (Γ_2^{IC}, helical) components of the cryptomelane magnetic structure, from the monoclinic-cell magnetic Rietveld refinements at 47, 44, and 40 K [Figures 9, S32, and S33]. Also given are the refined moments of the Mn_3O_4 impurity phase included at 40 K, for its three symmetry-inequivalent Mn sites, labeled following the sublattice notation of Jensen and Nielsen [14]: the tetrahedral Mn^{2+} site (A), and the two octahedral Mn^{3+} sites that are equivalent in the paramagnetic tetragonal cell but become crystallographically distinct once the ordered moment lowers the magnetic symmetry, distinguished according to whether accommodating a canted moment on that site requires doubling the magnetic cell (B_{d}) or not (B_{nd}). All three sites refine to moments collinear along the b-axis of the Mn_3O_4 ($I4_1/amd$) cell, consistent with the present (40 K) refinement lying above Jensen and Nielsen's cell-doubling transition at ∼33 K [14]; no Mn_3O_4 magnetic phase was included at 47 or 44 K.

	**Cryptomelane**		**Mn_3O_4**		
T (K)	Commensurate (μ_{B})	Incommensurate (μ_{B})	A (μ_{B})	B_{nd} (μ_{B})	B_{d} (μ_{B})
47	0.320(16)	0.513(4)	–	–	–
44	0.446(16)	0.596(4)	–	–	–
40	0.496(20)	0.701(4)	0.47(6)	0.68(5)	0.96(9)

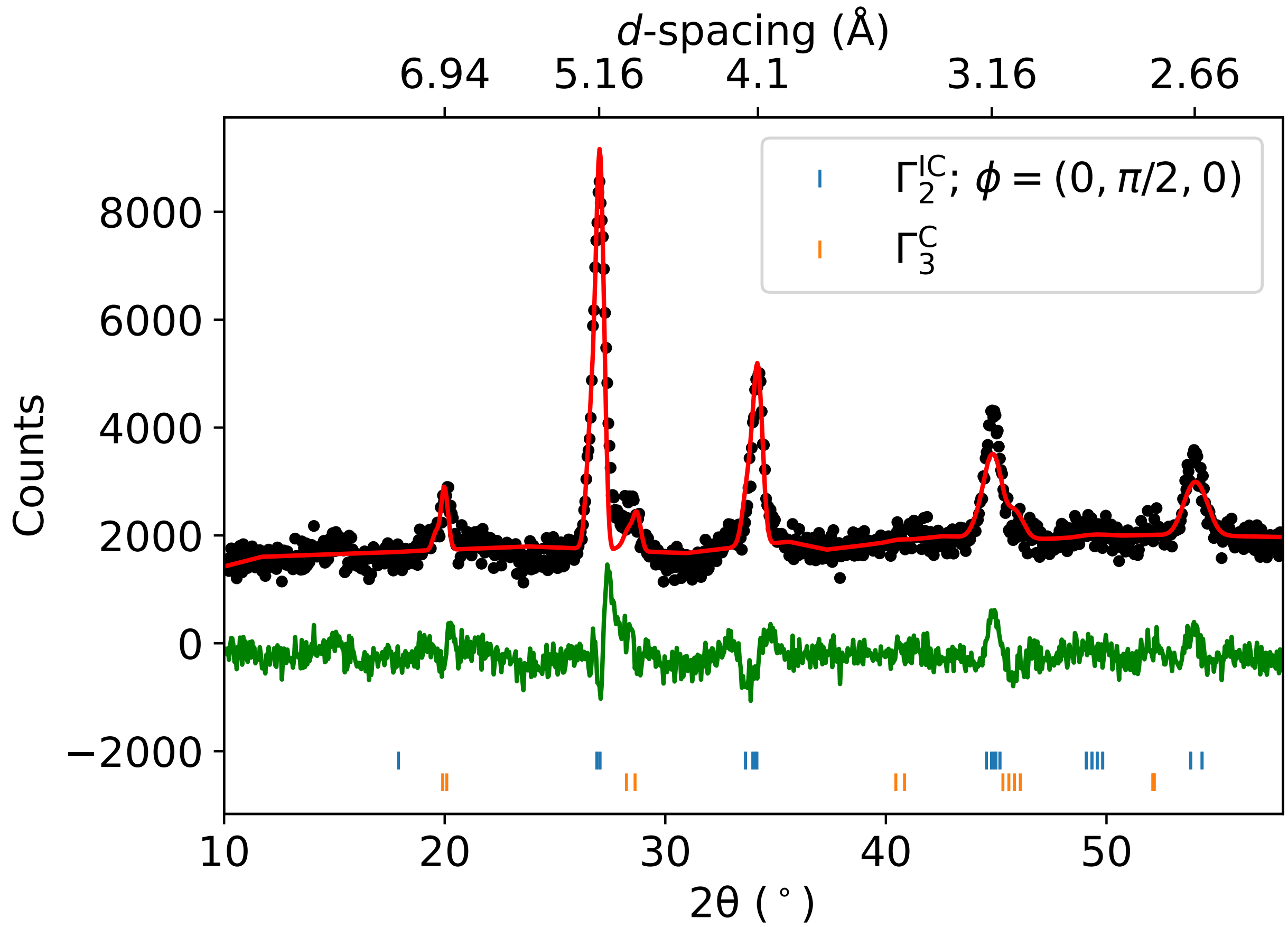

Figure S32:

Experimental difference pattern 44 K-60 K (measured on KMO-S1), compared with calculated pattern. The calculated pattern was obtained by Rietveld refinement of the same model as Figure 9, consisting of a commensurate $\vec{k}_{\mathrm{mag}} = 0$ (Γ_3^{C}) and incommensurate $\vec{k}_{\mathrm{mag}} = (0, 0, \sim 0.37)$ (Γ_2^{IC}, helical) component, assuming a monoclinic ($A2/m$) unit cell.

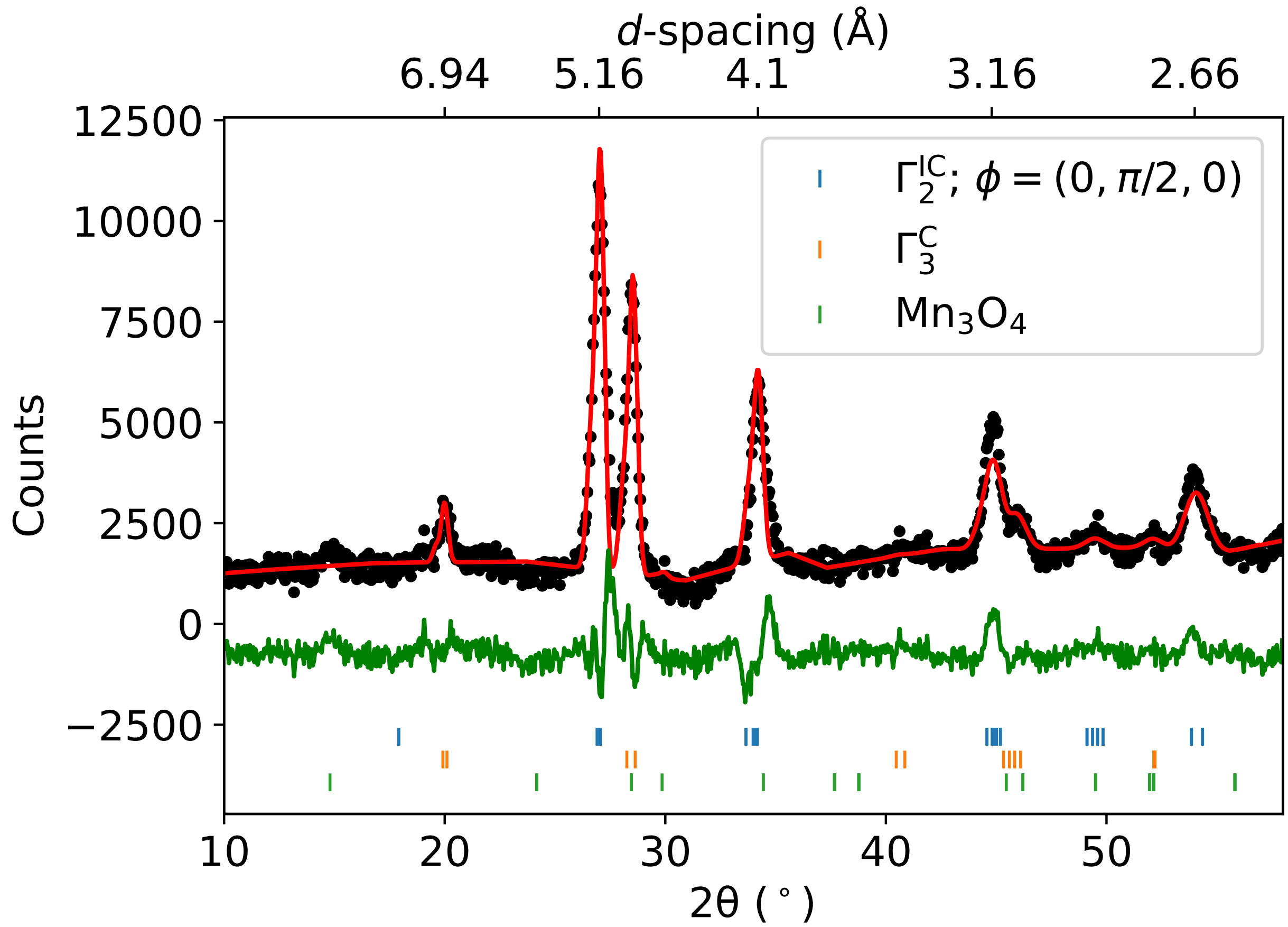

Figure S33: Experimental difference pattern 40 K-60 K (measured on KMO-S1), compared with calculated pattern. The calculated pattern was obtained by Rietveld refinement of the same model as Figure 9, consisting of a commensurate $\vec{k}_{\mathrm{mag}} = 0$ (Γ_3^{C}) and incommensurate $\vec{k}_{\mathrm{mag}} = (0, 0, \sim 0.37)$ (Γ_2^{IC}, helical) component, assuming a monoclinic ($A2/m$) unit cell, together with an additional magnetic phase for the Mn_3O_4 impurity ($\vec{k}_{\mathrm{mag}} = 0$, entirely collinear).

9. MAGNETIC NEUTRON DIFFRACTION ANALYSIS AT 47 K ASSUMING TETRAGONAL CELL

We have shown using synchrotron diffraction that there is a subtle transition to a monoclinic structure below T_1 in cryptomelane of the stoichiometry we study in this work. However, from our neutron diffraction data measured on D20, we are not actually able to distinguish between the monoclinic ($A2/m$) and tetragonal ($I4/m$) unit cells. In the main text of the paper, we present the analysis of the magnetic neutron diffraction data using the monoclinic cell. Here, we present alternative analysis of the same data using the tetragonal unit cell, to show that the conclusions we obtain using the highest-symmetry cell consistent with the data gives consistent results to the analysis with the monoclinic cell.

Indexing of the magnetic cell of cryptomelane in the temperature range $T_3 < T < T_2$, assuming a tetragonal cell, could not be carried out using a single propagation vector and instead 2 vectors, one commensurate and one incommensurate, were required. This was the case for the monoclinic cell also. We used $\vec{k}_{\mathrm{mag}} = (0, 0, 0)$ and $\vec{k}_{\mathrm{mag}} = (0, 0, k_z)$ [$k_z = 0.36902(15)$]. Only one of the main magnetic peaks, at $d = 6.95$ Å, is assigned to the commensurate magnetic structure. This peak corresponds to the nuclear [110] Bragg peak. As neutrons scatter from magnetic moment perpendicular to the scattering direction, this peak indicates that the magnetic moments are along a direction perpendicular to this vector. The most likely direction, based on the symmetry of the crystallographic unit cell, is either the [001] or [$\bar{1}$10] directions. Previous work on $K_{1.5}(H_3O)_xMn_8O_{16}$ single crystals indicates the magnetic hysteresis is much stronger in the *ab*-plane than along the *c* direction [15], which suggests that the moment is along [$\bar{1}$10]. The remaining four peaks ($d = 5.15, 4.10, 3.15, 2.65$ Å) can only be indexed to a propagation vector incommensurate with the crystal structure, $\vec{k}_{\mathrm{mag}} = (0, 0, \sim 0.37)$.

For each propagation vector, there are multiple symmetry-allowed IRs [Tables S7 and S8]. The correct IR would describe the basis vector behaviors at each magnetic site, which collectively make up the magnetic structure. There are 4 IRs for $\vec{k}_{\mathrm{mag}} = (0, 0, \sim 0.37)$ [Table S7] and 6 IRs for $\vec{k}_{\mathrm{mag}} = (0, 0, 0)$ [Table S8]; these are denoted using Γ_i^j ($i = 1, 2, 3, 4, 5,$ or 6; j = IC or C where IC/C stands for InCommensurate or Commensurate).

We use magnetic Rietveld refinement to test the IRs against the experimental difference pattern (47 K-60 K), shown in Figure S34 and determine the magnetic structure. There are

two IRs for the commensurate $\vec{k}_{\mathrm{mag}} = (0, 0, 0)$ component which successfully fit the data: Γ_1^{C}, with a single basis vector ψ_1, and Γ_3^{C} with four basis vectors. The refined unit cell for Γ_1^{C} consists of ferromagnetically-aligned Mn spins along the *c*-axis. A net moment along *c* is inconsistent with field-dependent magnetization measurements on single crystals [15] and so Γ_1^{C} is not considered further. Due to reasons of symmetry, there are a range of possible spin orientations in the *ab*-plane within Γ_3^{C}, both collinear and non-collinear, that are indistinguishable from powder neutron diffraction data [Figure S36]. The simplest of these is a ferromagnetic structure with moments aligned along the *a*- or *b*-axes, whereas more complex non-collinear ferromagnetic structures are also possible. As we cannot distinguish between them, we assume the simplest such structure as given in Figure S35(a,c).

For the incommensurate $\vec{k}_{\mathrm{mag}} = (0, 0, 0.37)$, it is possible to obtain a reasonable fit to the incommensurate peaks in the experimental diffraction data with Γ_i^{IC} (i=2,3,4) for several different models. The best fit is obtained with a helical model in which there is a constant moment in the *ab*-plane and a sinusoidally oscillating moment in the *c*-direction, which is slightly better than a similar fit with only the helical component in the *ab*-plane. These can be obtained using either Γ_3^{IC} and Γ_4^{IC}, with phase shifts (discussed in Section 7.3) applied in certain crystallographic directions. Γ_2^{IC} can also be used to fit the data, but with lower quality than Γ_3^{IC} and Γ_4^{IC}.

Given the correspondence with theoretical [16] and experimental [15, 17] works, we assume the structure is this *ab*-helical model, in which there are no moments along the *c*-axis and the moment is constant and rotating within the *ab*-plane. The Rietveld refinement at 47 K using the *ab*-helical model in conjunction with the collinear ferromagnetic Γ_3^{C} is shown in Figure S34, and the associated magnetic structure is shown in Figure S35(b).

We have explored the possibility of magnetic phase segregation into two phases each with one of these two IRs, by varying the relative scale factors, S_{C} and S_{IC}, of the two phases, but we find no strong evidence for this. Additionally, given that the sample has multivalent Mn, magnetic phase segregation would probably correspond to segregation of Mn^{3+} and Mn^{4+} which would be identifiable from structural analysis (as would the corresponding change in local K^+ content). We therefore consider it likely that both IRs coexist over the bulk of the sample. Fixing the scale factors $S_{\mathrm{C}} = S_{\mathrm{IC}} = S_{\mathrm{crystal}}$ the refinement determines the magnitude of each moment at 47 K for the commensurate component to be 0.337(17) μ_{B}, and the magnitude of the individual moments in the incommensurate component (assuming purely

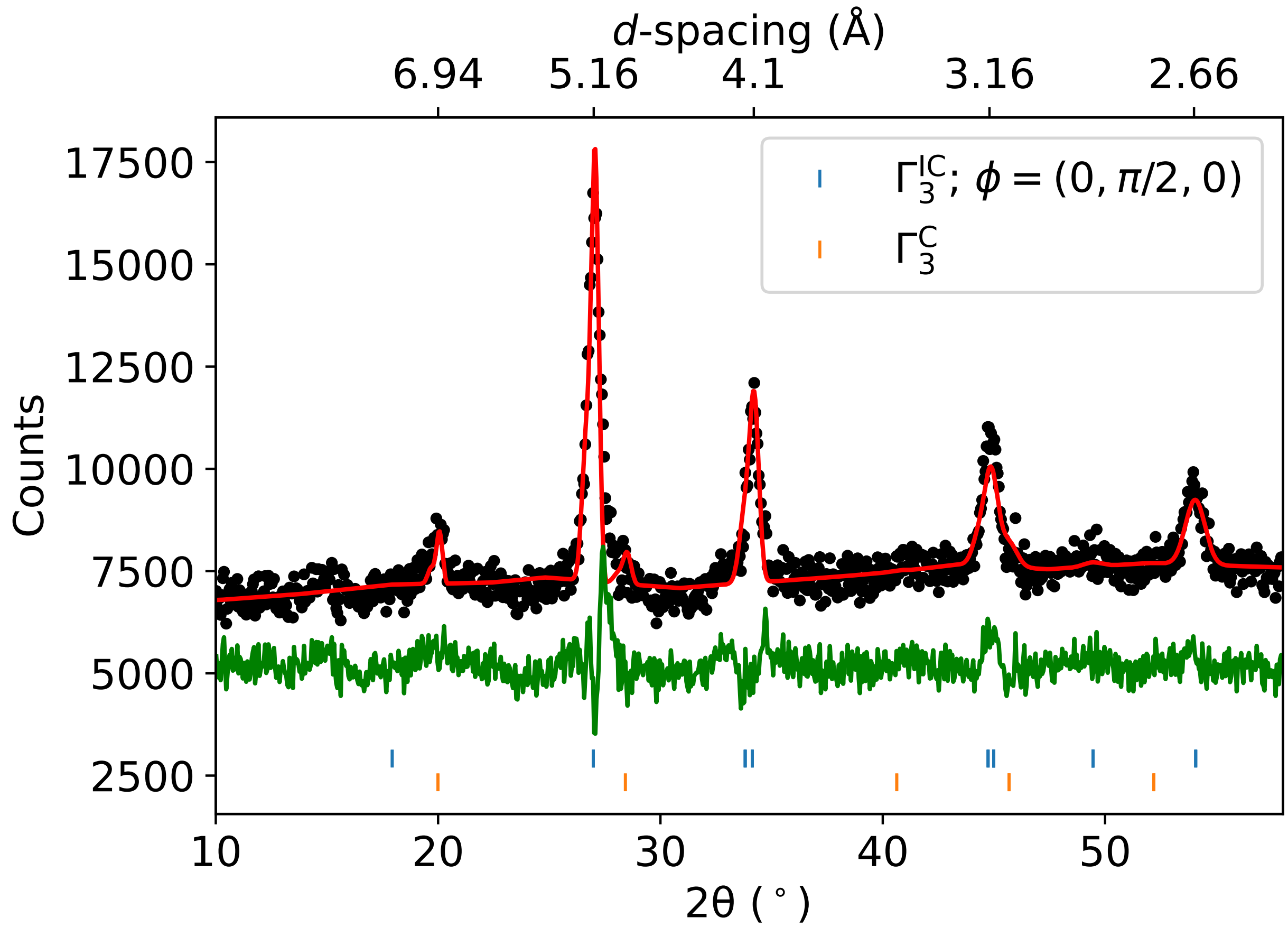

Figure S34: Experimental difference pattern 47 K-60 K (measured on KMO-S1), compared with calculated pattern. The calculated pattern was obtained by Rietveld refinement of the model for $T_3 < T < T_2$, consisting of a commensurate $\vec{k}_{\rm mag} = 0$ and incommensurate $\vec{k}_{\rm mag} = (0, 0, \sim 0.37)$ component, assuming a tetragonal ($I4/m$) unit cell. The commensurate structure was represented by that given in Figure S36(a). The incommensurate structure was represented with a helical model, with moments in the ab-plane.

helical modulation) to be $0.503(4)\,\mu_{\rm B}$. The net magnitude of each individual moment would result from the superposition of the commensurate and incommensurate components; at 47 K, the magnitude for each moment occurs in the range $0.503(4){\pm}0.337(17)\,\mu_{\rm B}$. We note here that at 47 K the magnetization is far from being saturated as can be clearly seen from Figure 4(a), which is why the moment magnitudes are smaller than the fully saturated moments expected for Mn^{3+}/Mn^{4+}. The obtained moment from the commensurate component is approximately consistent with that obtained from the fixed-temperature, variable-field

measurement [Figure 6 of main text].

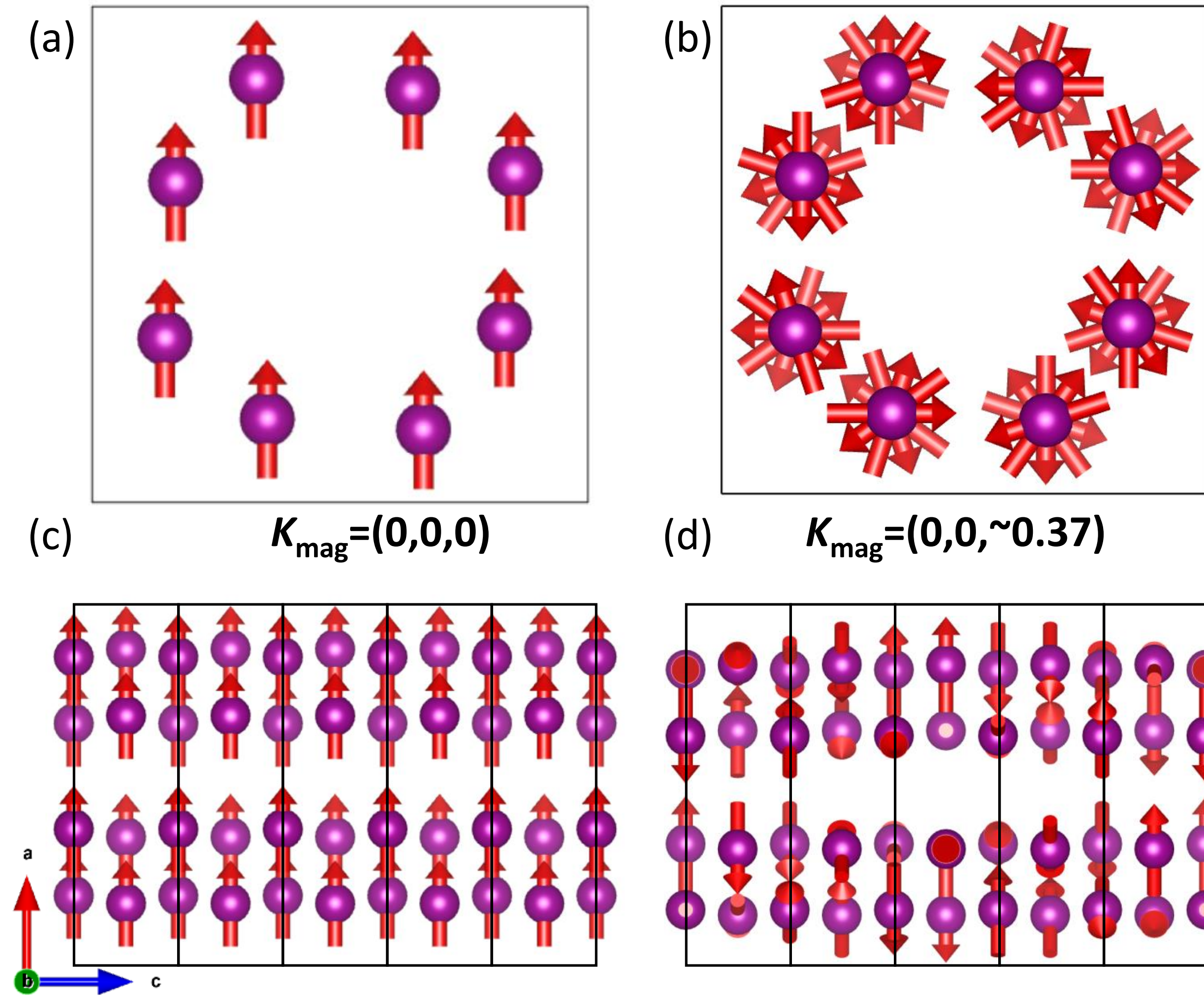

Figure S35: The refined magnetic unit cell for the (a,c) commensurate $\vec{k}_{\mathrm{mag}} = (0,0,0)$ and (b,d) incommensurate $\vec{k}_{\mathrm{mag}} = (0,0,0.37)$ components of the magnetic structure for $T_3 < T < T_2$ assuming a tetragonal ($I4/m$) unit cell. In (a,c), we assume the commensurate component is entirely ferromagnetic, but we note that the data would also be consistent with a net moment in any orientation within the ab-plane, including non-collinear arrangements [Figure S36]. In (b,d), we assume the modulated magnetism is helical, with the moment entirely within the ab-plane, but we note that a sinusoidal model also fits the data.

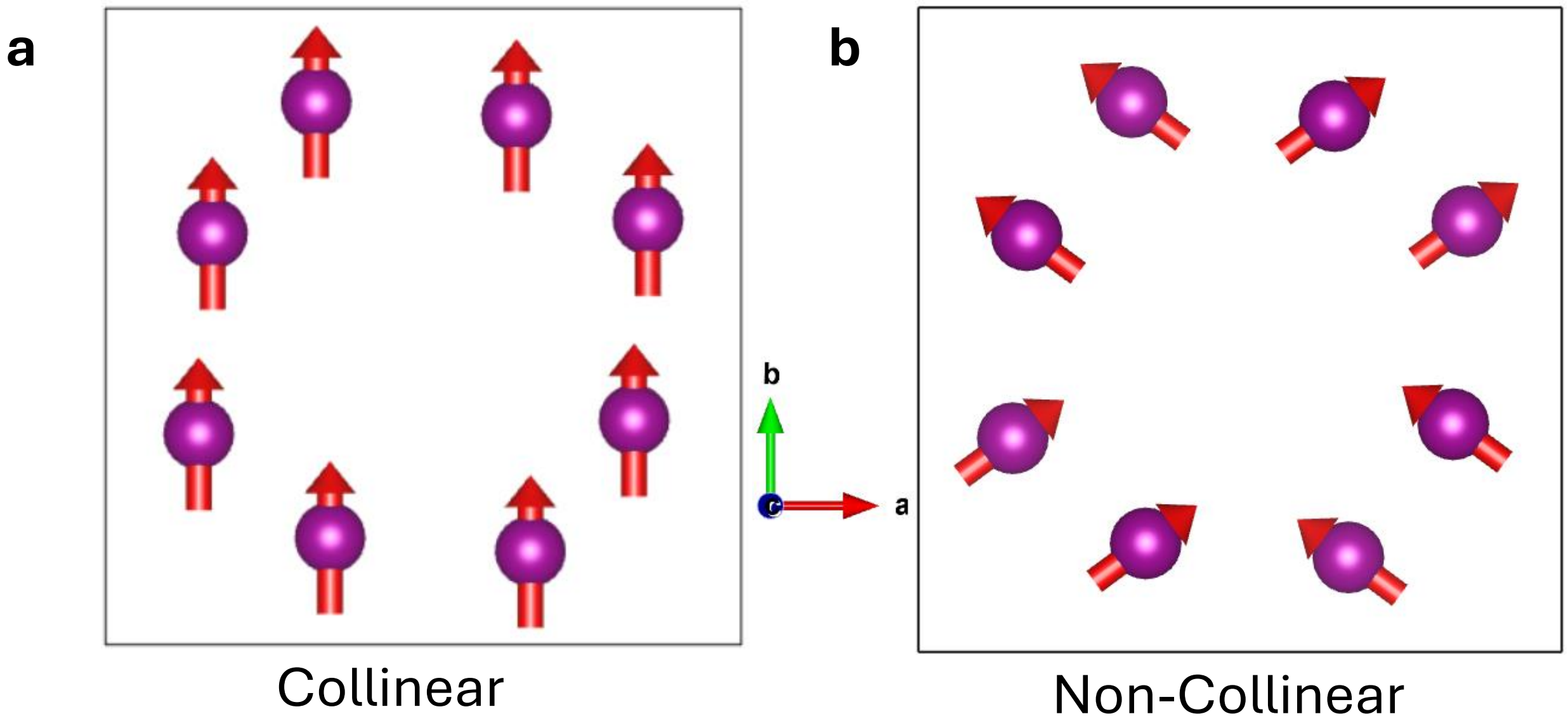

Figure S36: Possible commensurate components of the magnetic structure for $T_3 < T < T_2$ assuming a tetragonal ($I4/m$) unit cell. Due to the symmetry of the unit cell, it is not possible to distinguish between a range of possible ferromagnetic cells including (a) collinear and (b) non-collinear cells (setting the constraints $C_1 = C_4$ and $C_2 = C_3$). The non-collinear component resembles a model proposed in theoretical work using Ising spins and described as "C2-AFM" [18].

10. MAGNETOMETRY ON KMO-S2

As noted elsewhere, most measurements presented in this manuscript are on one batch of cryptomelane, KMO-S1 (Table S1). However the variable-temperature synchrotron diffraction was not collected on the same batch, but on a separately-synthesized batch, KMO-S2. We have collected magnetometry data on KMO-S2, which we show in Figure S37. This shows similar magnetic behavior to KMO-S1, but the transition T_1 occurs at a slightly different, and higher, temperature, as shown in Table S10. This modest difference in T_1, together with the differing secondary-phase weight fractions of the two batches (Table S2), indicates some batch-to-batch variation in composition and/or microstructure, notwithstanding the closely comparable room-temperature lattice parameters of KMO-S1 and KMO-S2 discussed in Section 1.

TABLE S10: Transition temperatures T_1, T_2, and T_3 for both cryptomelane batches studied in this work. Where values are determined from magnetometry, the maximum in $d\chi/dT$ is used where possible, though very broad point spacing in the KMO-S2 data means some intuition is employed. For T_1, values are given separately as determined from diffraction and from magnetometry; the diffraction value is roughly estimated based on the point at which pseudo-tetragonal lattice parameters stabilize above the transition [Figure S13].

		**KMO-S1**	**KMO-S2**
T_1 (K)	Diffraction	N/A	∼175
	Magnetometry	184	185
T_2 (K)		54.5	56[a]
T_3 (K)		24	22

[a] Note this feature is weakly resolved due to the poor point spacing of these data.

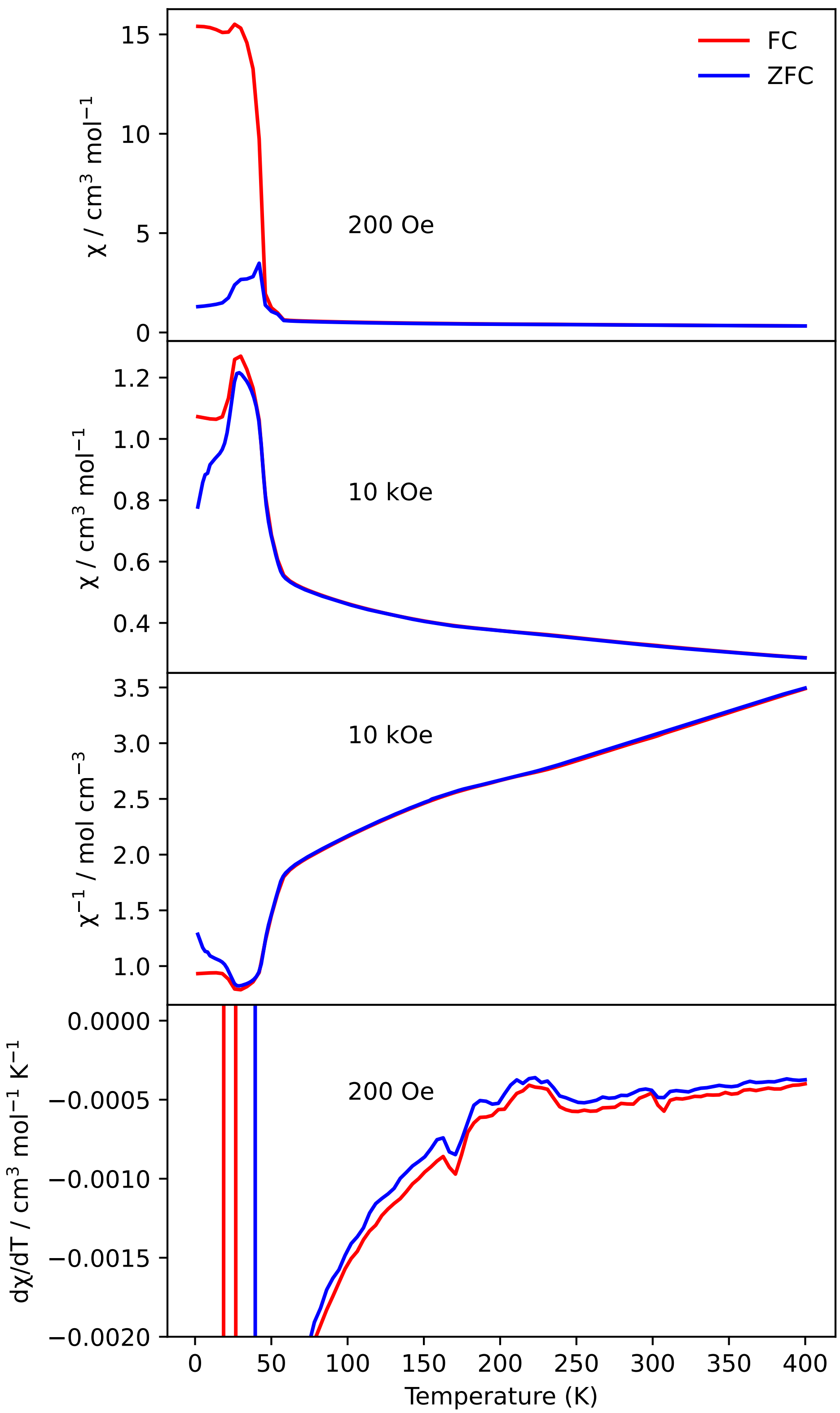

Figure S37: Magnetometry on KMO-S2.

11. CURIE-WEISS ON KMO-S1

Fitting the Curie-Weiss law [19] to data which was measured on heating in a field of 200 Oe, in the range 200 K to 300 K yields effective moment μ_{eff} =5.00(4) μ_{B} for the ZFC-W susceptibility data and 4.80(3) μ_{B} for the FC-W susceptibility data, along with Weiss constants of -483(6) K and -550(10) K, respectively. This effective moment exceeds what we would expect for a mixed-$Mn^{3+/4+}$ system with this stoichiometry ($\sim 4.1\,\mu_{\text{B}}$).

We caution that this analysis has significant limitations. The 200–300 K fitting range is not demonstrably within the high-temperature paramagnetic regime, since it lies below the modulus of the fitted Weiss constant (and the constant reported by other works, such as Larson [20]); below the Weiss temperature, persistent local magnetic correlations are expected to cause deviations from ideal Curie-Weiss behavior. Additionally, the ZFC-W and FC-W susceptibility data partially diverge in this temperature range (as shown in Figure 4(b) of the main text), which would not be expected for a system obeying the Curie-Weiss law. Finally, susceptibility measured as a function of applied field at 100, 150, and 300 K shows a very subtle non-linearity at low field ($< 500\,Oe$), consistent with a small contribution from trace magnetic ions; this would tend to inflate the apparent susceptibility at 200 Oe, and hence the extracted μ_{eff} and Weiss temperature, obtained from measurements at 200 Oe. Given these limitations, the values obtained here should not be relied upon quantitatively.

11.1. Error propagation

In this paper, we present the Weiss temperature and μ_{eff} per Mn as obtained by fitting to the variable-temperature magnetic susceptibility data the Curie-Weiss law [19]:

$$\chi = \frac{C}{T - T_0} \tag{5}$$

where C is the Curie constant and T_0 is the Weiss constant. Fits are performed by taking the inverse of the susceptibility data and fitting an order-1 polynomial to the data using NumPy [3]. During the fit, each data-point is assigned a weighting, which is the inverse of its error assuming all the error in the measurement comes from uncertainty in the sample mass, due to a weighing precision of 0.1 mg. Uncertainty in the gradient and y-intercept of this linear fit come from the diagonal elements of the resulting matrix of covariance, and by

standard error propagation these are used to obtain errors in C and T_0.

[1] M. L. Carter and R. L. Withers, A universally applicable composite modulated structure approach to ordered $Ba_xM_yTi_{8-y}O_{16}$ hollandite-type solid solutions, Journal of Solid State Chemistry **178**, 1903 (2005).

[2] D. R. Smith and F. R. Fickett, Low-temperature properties of silver, Journal of Research of the National Institute of Standards and Technology **100**, 119 (1995).

[3] C. R. Harris, K. J. Millman, S. J. van der Walt, R. Gommers, P. Virtanen, D. Cournapeau, E. Wieser, J. Taylor, S. Berg, N. J. Smith, R. Kern, M. Picus, S. Hoyer, M. H. van Kerkwijk, M. Brett, A. Haldane, J. F. del Río, M. Wiebe, P. Peterson, P. Gérard-Marchant, K. Sheppard, T. Reddy, W. Weckesser, H. Abbasi, C. Gohlke, and T. E. Oliphant, Array programming with NumPy, Nature **585**, 357 (2020).

[4] P. Debye, Zur theorie der spezifischen wärmen, Annalen der Physik **344**, 789 (1912).

[5] P. Virtanen, R. Gommers, T. E. Oliphant, M. Haberland, T. Reddy, D. Cournapeau, E. Burovski, P. Peterson, W. Weckesser, J. Bright, S. J. van der Walt, M. Brett, J. Wilson, K. J. Millman, N. Mayorov, A. R. J. Nelson, E. Jones, R. Kern, E. Larson, C. J. Carey, İ. Polat, Y. Feng, E. W. Moore, J. VanderPlas, D. Laxalde, J. Perktold, R. Cimrman, I. Henriksen, E. A. Quintero, C. R. Harris, A. M. Archibald, A. H. Ribeiro, F. Pedregosa, P. van Mulbregt, and SciPy 1.0 Contributors, SciPy 1.0: Fundamental Algorithms for Scientific Computing in Python, Nature Methods **17**, 261 (2020).

[6] P. W. Stephens, Phenomenological model of anisotropic peak broadening in powder diffraction, Journal of Applied Crystallography **32**, 281 (1999).

[7] W. H. Baur, The geometry of polyhedral distortions. Predictive relationships for the phosphate group, Acta Crystallographica Section B: Structural Crystallography and Crystal Chemistry **30**, 1195 (1974).

[8] L. A. V. Nagle-Cocco and S. E. Dutton, Van Vleck analysis of angularly distorted octahedra using VanVleckCalculator, Journal of Applied Crystallography **57(1)**, 20 (2024).

[9] J. H. Van Vleck, The Jahn-Teller effect and crystalline Stark splitting for clusters of the form XY_6, The Journal of Chemical Physics **7**, 72 (1939).

[10] J. Kanamori, Crystal distortion in magnetic compounds, Journal of Applied Physics **31**, S14 (1960).

[11] J. Rodríguez-Carvajal, A program for calculating irreducible representation of little groups and basis functions of polar and axial vector properties, Program included FullProf Suite, version July-2010, ILL (2010).

[12] O. V. Kovalev, *Representations of the Crystallographic Space Groups: Irreducible Representations, Induced Representations, and Corepresentations* (Gordon and Breach Science, 1993).

[13] J. Rodríguez-Carvajal, Fullprof, CEA/Saclay, France (2001).

[14] G. B. Jensen and O. V. Nielsen, The magnetic structure of Mn_3O_4 Hausmannite between 4.7 K and Neel point, 41 K, Journal of Physics C: Solid State Physics **7**, 409 (1974).

[15] H. Sato, T. Enoki, J.-I. Yamaura, and N. Yamamoto, Charge localization and successive magnetic phase transitions of mixed-valence manganese oxides $K_{1.5}(H_3O)_xMn_8O_{16}$ ($0< x <0.5$), Physical Review B **59**, 12836 (1999).

[16] S. Mandal, A. Andreanov, Y. Crespo, and N. Seriani, Incommensurate helical spin ground states on the hollandite lattice, Physical Review B **90**, 104420 (2014).

[17] H. Sato, J.-I. Yamaura, T. Enoki, and N. Yamamoto, Magnetism and electron transport phenomena of manganese oxide ion exchanger with tunnel structure, Journal of Alloys and Compounds **262**, 443 (1997).

[18] Y. Crespo, A. Andreanov, and N. Seriani, Competing antiferromagnetic and spin-glass phases in a hollandite structure, Physical Review B **88**, 014202 (2013).

[19] S. Mugiraneza and A. M. Hallas, Tutorial: a beginner's guide to interpreting magnetic susceptibility data with the Curie-Weiss law, Communications Physics **5**, 95 (2022).

[20] A. M. Larson, *Frustrated Magnetism and Electronic Properties of Hollandite Oxide Materials (Chapter 4)*, Ph.D. thesis, University of Maryland (2017).
